# Publisher Correction: Dynamic and static control of the off-target interactions of antisense oligonucleotides using toehold chemistry

**DOI:** 10.1038/s41467-023-44590-4

**Published:** 2024-01-04

**Authors:** Chisato Terada, Kaho Oh, Ryutaro Tsubaki, Bun Chan, Nozomi Aibara, Kaname Ohyama, Masa-Aki Shibata, Takehiko Wada, Mariko Harada-Shiba, Asako Yamayoshi, Tsuyoshi Yamamoto

**Affiliations:** 1 Department of Chemistry of Biofunctional Molecules, Graduate School of Biomedical Sciences, Nagasaki University, Nagasaki, Japan; 2 JSPS Research Fellow (DC1), Japan Society for the Promotion of Science, Tokyo, Japan; 3 Graduate School of Engineering, Nagasaki University, Nagasaki, Japan; 4 Department of Pharmacy Practice, Graduate School of Biomedical Sciences, Nagasaki University, Nagasaki, Japan; 5 Department of Molecular Pathochemistry, Graduate School of Biomedical Sciences, Nagasaki University, Nagasaki, Japan; 6 Department of Anatomy and Cell Biology, Faculty of Medicine, Osaka Medical and Pharmaceutical University, Takatsuki, Japan; 7 Institute of Multidisciplinary Research for Advanced Materials (IMRAM), Tohoku University, Sendai, Miyagi Japan; 8 Department of Molecular Innovation in Lipidology, National Cerebral and Cardiovascular Center Research Institute, Suita, Japan; 9 Cardiovascular Center, Osaka Medical and Pharmaceutical University, Takatsuki, Japan

**Keywords:** Antisense oligonucleotide therapy, Peptide nucleic acid oligo, Nanobiotechnology, Gene regulation

Correction to: *Nature Communications* 10.1038/s41467-023-43714-0, published online 02 December 2023

In this article, Fig. 3e did not display correctly in the PDF and HTML. The original article has been corrected.

