# Supplementary material for: Dynamic and static control of the off-target interactions of antisense oligonucleotides using toehold chemistry
Source: Nat Commun. 2023 Dec 2;14:7972. doi: 10.1038/s41467-023-43714-0 (PMC10693639; doi:10.1038/s41467-023-43714-0)
Supplement: Supplementary file 1 — Supplementary Information [file 41467_2023_43714_MOESM1_ESM.pdf]

Supplementary information

Dynamic and static control of the off-target interactions of antisense oligonucleotides using toehold chemistry

Chisato Terada[1,2], Kaho Oh[1], Ryutaro Tsubaki[1], Bun Chan[3], Nozomi Aibara[4], Kaname Ohyama[5], Masa-Aki Shibata[6], Takehiko Wada[7], Mariko Harada-Shiba[8,9], Asako Yamayoshi[1], Tsuyoshi Yamamoto[1]*

[1]Chemistry of Biofunctional Molecules, Graduate School of Biomedical Sciences, Nagasaki University, Nagasaki, Japan

[2]JSPS Research Fellow (DC1), Japan Society for the Promotion of Science, Tokyo, Japan

[3]Graduate School of Engineering, Nagasaki University, Nagasaki, Japan

[4]Department of Pharmacy Practice, Graduate School of Biomedical Sciences, Nagasaki University, Nagasaki, Japan

[5]Department of Molecular Pathochemistry, Graduate School of Biomedical Sciences, Nagasaki University, Nagasaki, Japan

[6]Department of Anatomy and Cell Biology, Faculty of Medicine, Osaka Medical and Pharmaceutical University, Takatsuki, Japan

[7]Institute of Multidisciplinary Research for Advanced Materials (IMRAM), Tohoku University, Sendai, Miyagi, Japan

[8]Department of Molecular Innovation in Lipidology, National Cerebral and Cardiovascular Center Research Institute, Suita, Japan

[9]Cardiovascular Center, Osaka Medical and Pharmaceutical University, Takatsuki, Japan

Index

Supplementary Figure 1 3
Supplementary Figure 2 5
Supplementary Figure 3 8
Supplementary Figure 4 9
Supplementary Table 1 10
Supplementary Figure 5 10
Supplementary Figure 6 10
Supplementary Table 2 11

Supplementary Figure 7 11
Supplementary Figure 8 11
Supplementary Figure 9 12
Supplementary Figure 10 20
Supplementary Figure 11 21
Supplementary Figure 12 21
Supplementary Figure 13 22
Supplementary Table 3 23
Supplementary Figure 14 23
Supplementary Figure 15 24
Supplementary Figure 16 25
Supplementary Figure 17 26
Supplementary Table 4 27
Supplementary Table 5 28
Supplementary Table 6 29
Supplementary Table 7 30
Supplementary Figure 18 30
Supplementary Table 8 31

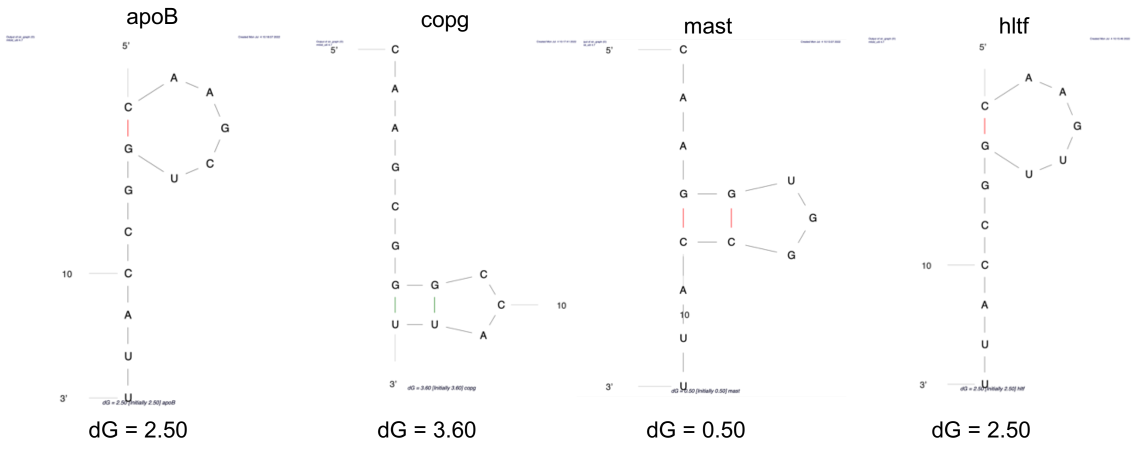

Supplementary Figure 1 The prediction of the secondary structure of each RNA (*apoB*, *copg*, *mast* and *hltf*) by mfold web server. (http://www.unafold.org/mfold/software/download-mfold.php)

		Degenerative changes*			The number of foci		The numer of TUNEL positive
		Zone1	Zone2	Zone3	Inflamation**	Microfocal necrosis of hepatocytes with cell infiltrations***	nuclears of hepatocytes
Saline	1	+	+	+	3	1	2
	2	+	+	+	2	0	1
	3	+	+	+	1	1	0
**mPCS1**	1		+	+	12	9	1
	2	+	+		26	13	8
	3		+	+	13	10	2
**mPCS1 /PNA(C8)**	1			+	8	6	2
	2		+	+	1	1	0
	3		+	++	3	4	1

*granular degeneration ～ hydropic degeneration; + mild, ++ moderate, +++severe

***Microfocal necrosis of hepatocytes with cell infiltrations

*** Eosinophilic and hyalinized hepatocytes

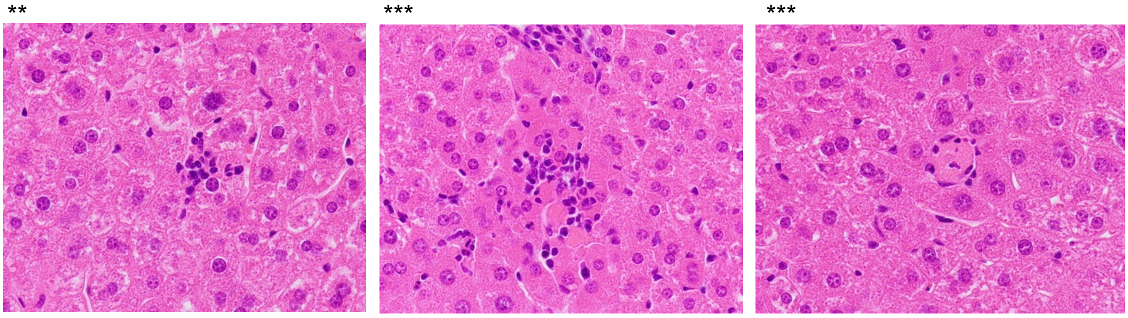

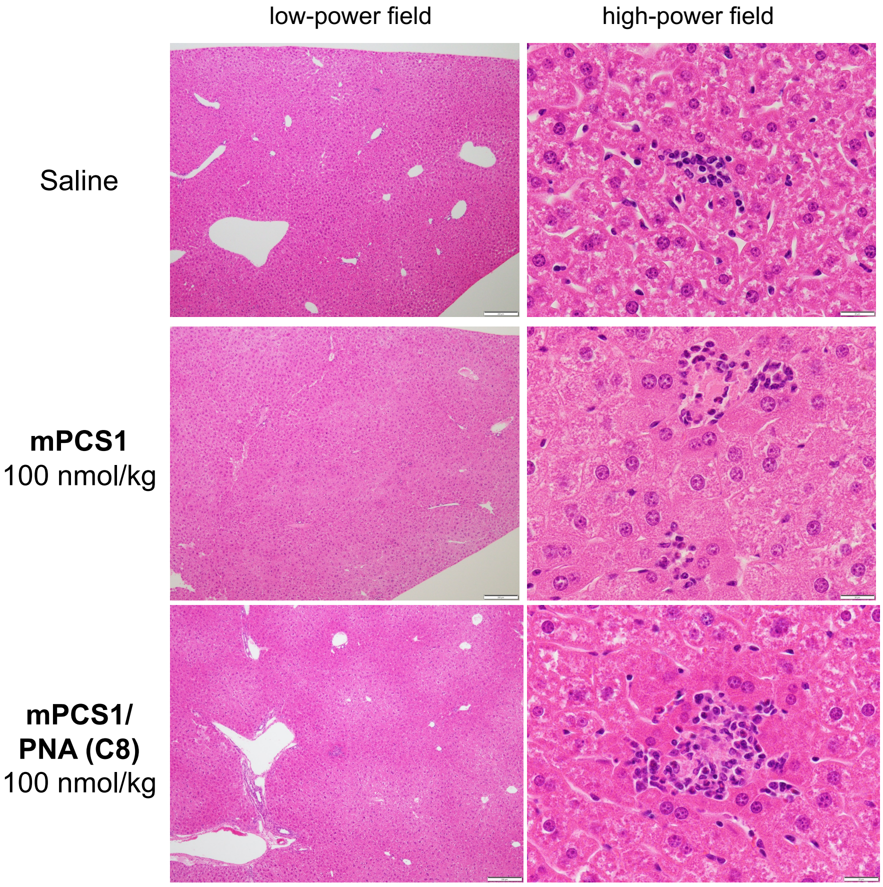

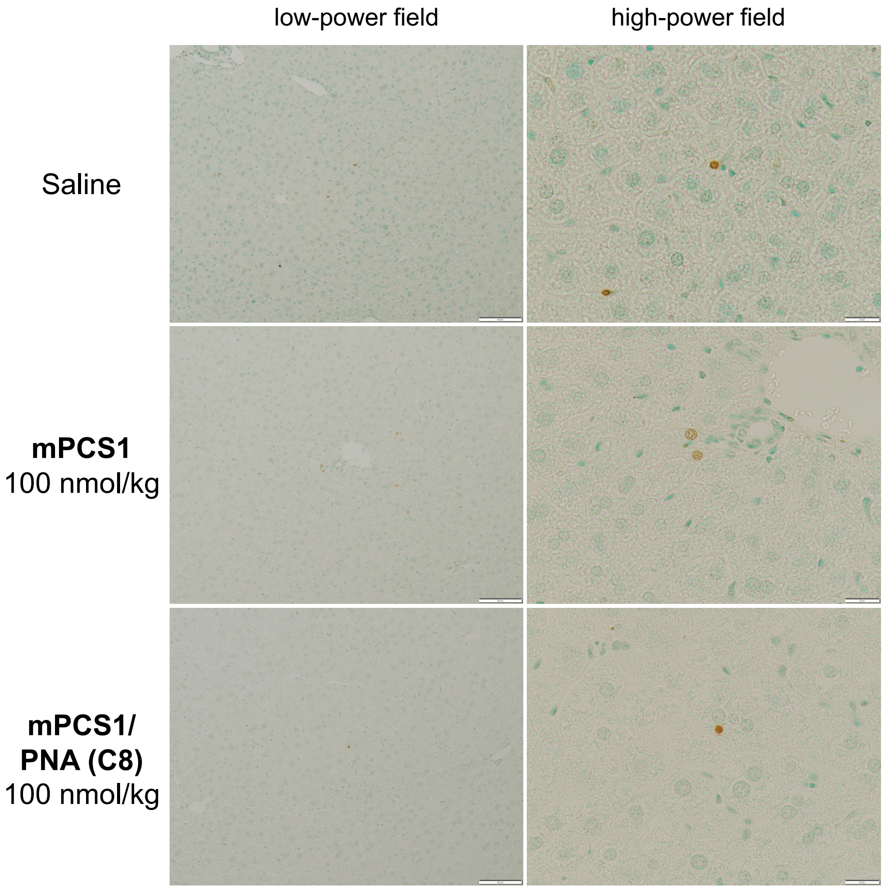

Supplementary Figure 2 Histopathological image analysis and representative images of TUNEL staining in livers of mice treated with saline, 100 nmol/kg **mPCS1** or **mPCS1/PNA(C8)** (*n* = 3 biologically independent samples).

		Degenerative changes*			The number of foci		
					Micro-focal aggregation of inf. cell	Necrosis of hepatocyte	
		Zone1	Zone2	Zone3		Focal	Micro-focal
Saline	1	++	+		2	-	1
	2	++	+		2	-	-
	3	++	+		2	-	-
**mPCS1**	1	++	+		2	39	-
	2	++	+		11	2	1
	3	++	+		16	-	10
**mPCS1 /PNA(C8)**	1	+			3	5	9
	2**	+	+	+	-	-	-
	3	+	+		1	-	1

* granular degeneration ～ hydropic degeneration; + mild, ++ moderate, +++severe

** No significant changes other than degeneration.

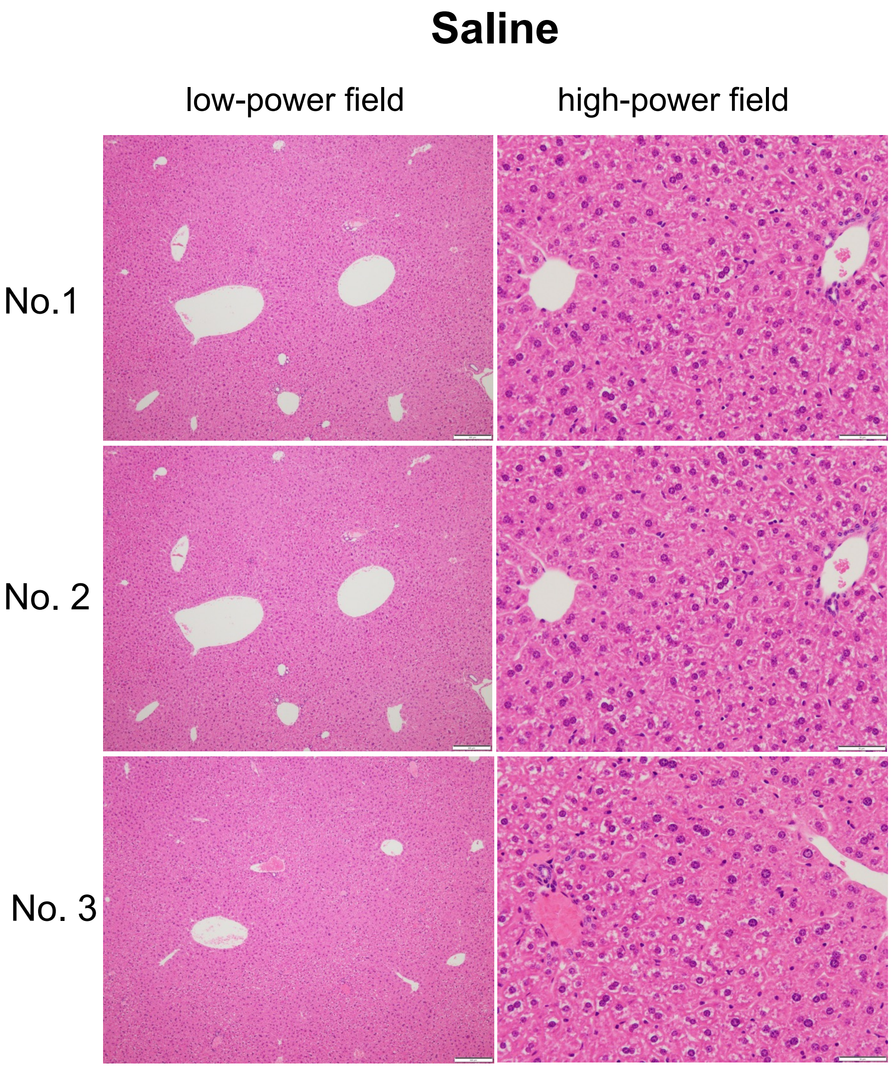

mPCS1

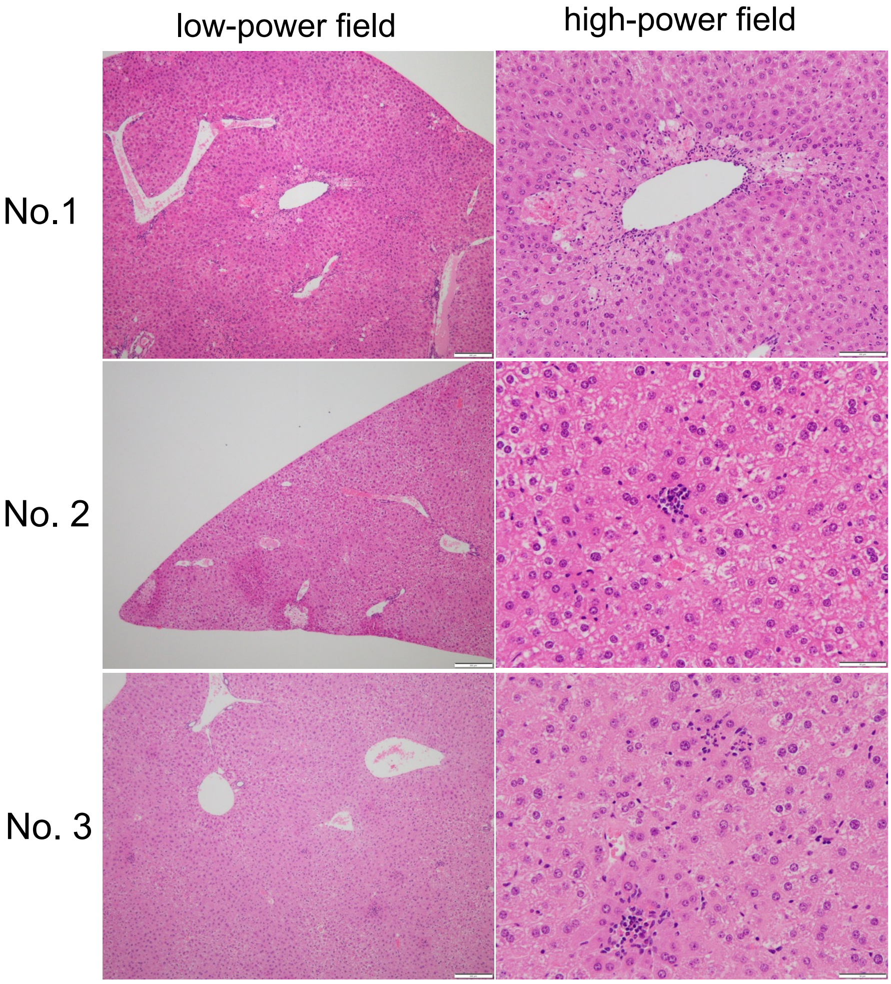

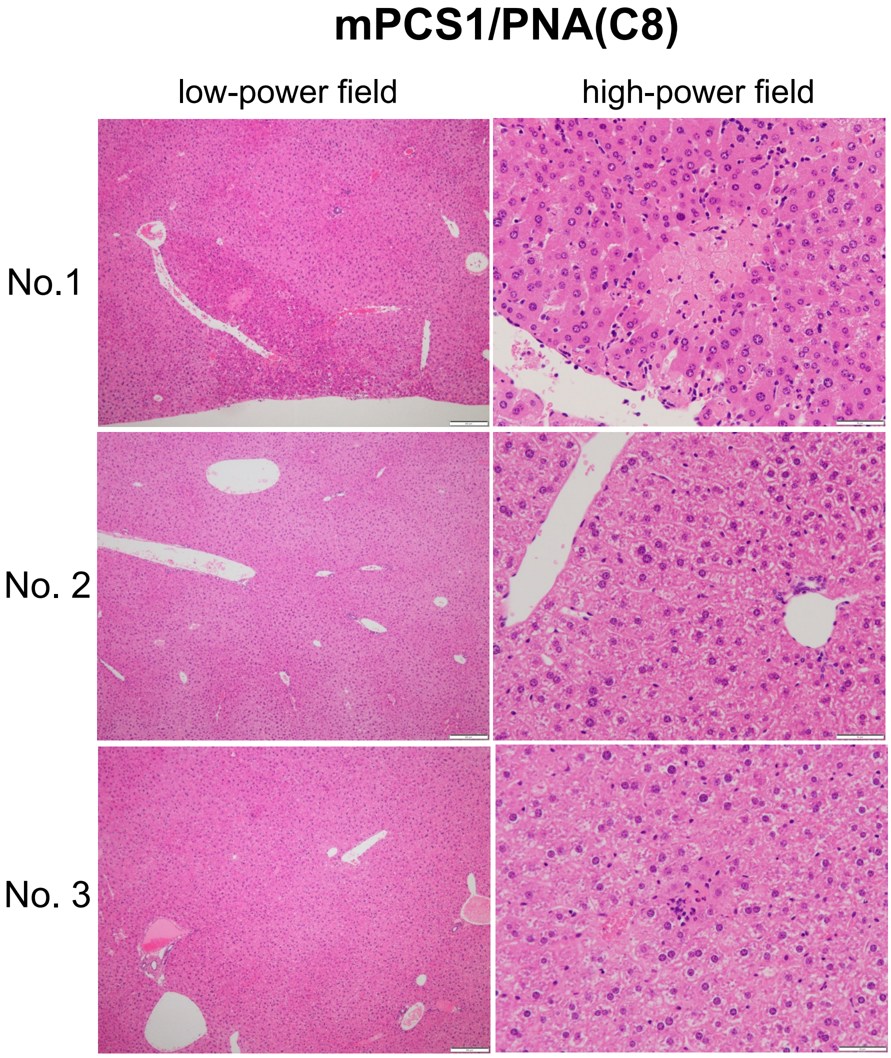

Supplementary Figure 3 Histopathological image analysis and images of H&E staining in livers of mice treated with saline, 200 nmol/kg **mPCS1** or **mPCS1/PNA (C8)**. (*n* = 3 biologically independent samples).

		Degenerative changes*			The number of foci	
		Zone1	Zone2	Zone3	Inflamation**	Microfocal necrosis of hepatocytes with cell infiltrations
Saline	1			+	1	-
	2			+	2	-
	3			+	-	-
**mPCS1**	1			+++	28	6
	2			+++	37	6
	3			+++	25	18
**mPCS1 /PNA(C8)**	1			+	17	3
	2***				7	-
	3***				3	1

*granular degeneration ～ hydropic degeneration; + mild, ++ moderate, +++severe

** Microfocal aggregation of inf. cells

***Small inflammatory clusters are observed, but lesions are not clearly defined.

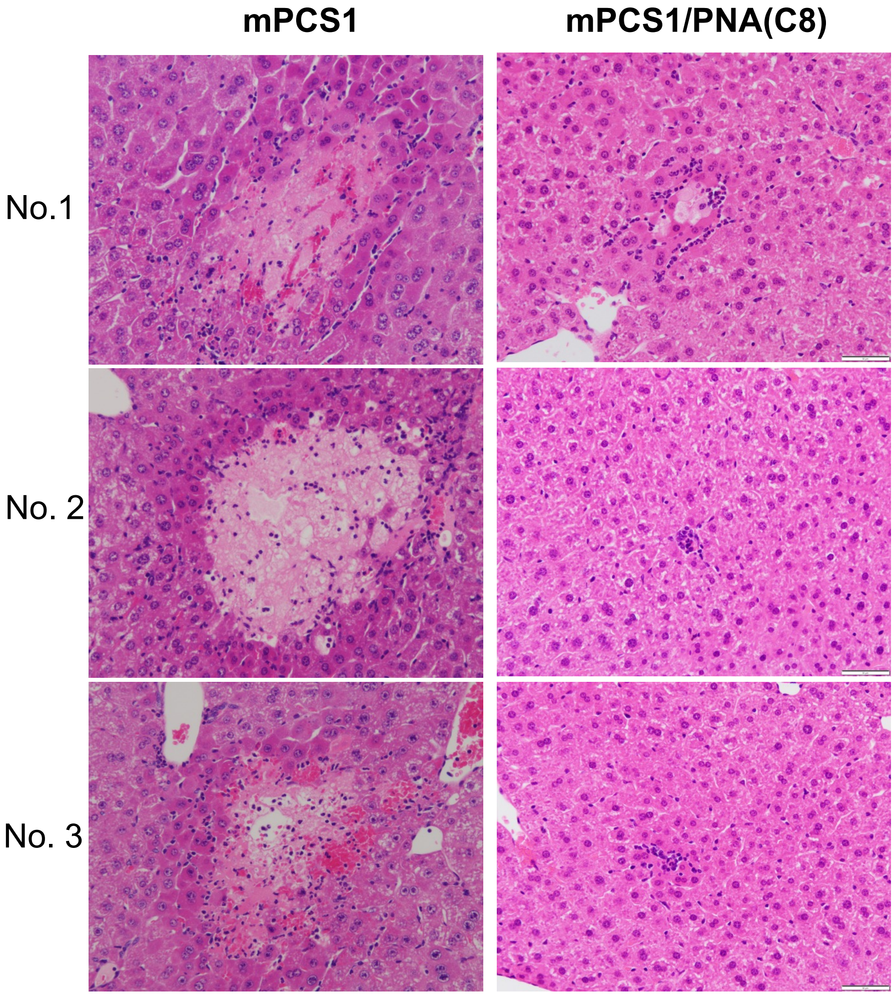

Supplementary Figure 4 Histopathological image analysis and conspicuous lesion area identified by H&E staining in livers of mice treated with saline, 400 nmol/kg **mPCS1** or **mPCS1/PNA(C8)**. (*n* = 3 biologically independent samples).

Supplementary Table 1. The number of change in gene expression as the results of introduction of **mPCS1** or **mPCS1/PNA (C8)**. *d* indicates the total number of mismatches, insertions, or deletions between the ASO and complementary RNA.

		$d = 0$	$d = 1$	$d = 2$	$d > 2$	Total
# of off-target (mouse)	Theoretical	5	250	6643	-	-
	Expressed	5	~219	~5526	~12315	~18065
# of DEG (Saline vs ssASO)	Up	0	6	255	695	956
	Down	4	50	467	666	1187
	Total	4	56	722	1361	2143
# of DEG (Saline vs BRO)	Up	0	0	81	220	301
	Down	2	15	107	137	261
	Total	2	15	188	357	562

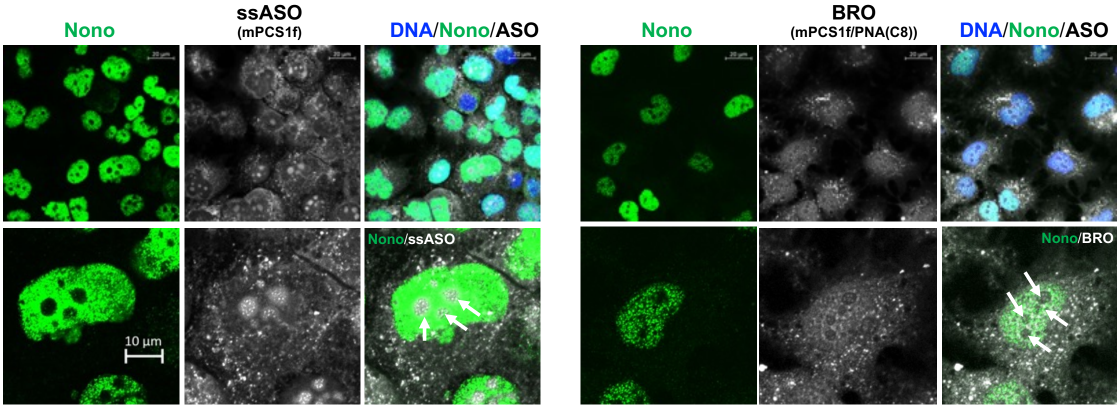

Supplementary Figure 5 Immunofluorescence staining of P54nrb in Huh-7 cells transfected with **mPCS1f** or **mPCS1f/PNA(C8)** at 250 nM for 48h. Note that Supplementary Fig.5 is based on limited trials with n=1 biologically independent samples.

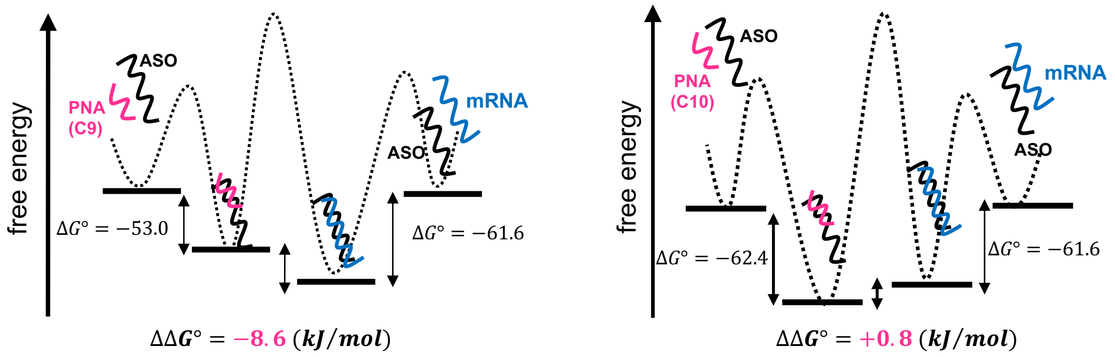

Supplementary Figure 6 Energy diagram of strand displacement based on experimentally determined ⊿G.

Supplementary Table 2. Screening of coupling conditions of H_2N-CAGT-$CONH_2$ (entries 1-7), H_2N-TTTT-$CONH_2$ (entry 8). H_2N-TTTTTT-$CONH_2$ (entries 9-10).

Entry	Resin	Activator	Base	Temperature	Equivalent (Monomer /Activator)	Coupling time	Crude yield (%)	HPLC purity [260 nm][a]
1	Fmoc-SAL-PEG	HATU	DIPEA	RT	3 eq	30 min	15	33
2	Fmoc-SAL-PEG	HATU	DIPEA	60 ℃	3 eq	30 min	5	trace
3	Fmoc-SAL-PEG	HATU	DIPEA	RT	5 eq	30 min	21	66
4	Fmoc-SAL-PEG	HATU/HOAt	DIPEA	RT	5 eq	30 min	10	58
5	Fmoc-SAL-PEG	HATU	DIPEA	RT	1) 3 eq<br>2) 2 eq	1) 15 min<br>2) 15 min	42	59
6	Fmoc-SAL-PEG	PyAOP	NMM	RT	1) 3 eq<br>2) 2 eq	1) 15 min<br>2) 15 min	55	87
7	Fmoc-SAL-PEG	HATU	NMM	RT	1) 3 eq<br>2) 2 eq	1) 15 min<br>2) 15 min	55	73
8	2-Chlorotrityl chloride	PyAOP	NMM	RT	1) 3 eq<br>2) 2 eq	1) 15 min<br>2) 15 min	63	43
9	Novasyn® TG	PyAOP	NMM	RT	1) 3 eq<br>2) 2 eq	1) 15 min<br>2) 15 min	18	26
10	Fmoc-Gly-TrtA-PEG	PyAOP	NMM	RT	1) 3 eq<br>2) 2 eq	1) 15 min<br>2) 15 min	26	64

[a] HPLC-based purity of crude PNA products measured at 260 nm.

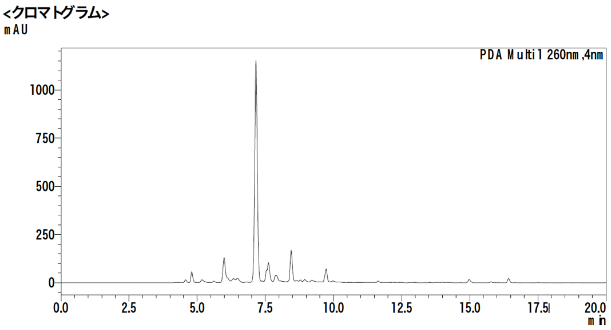

Supplementary Figure 7. HPLC analysis of HATU/DIPEA condition (entry 5).

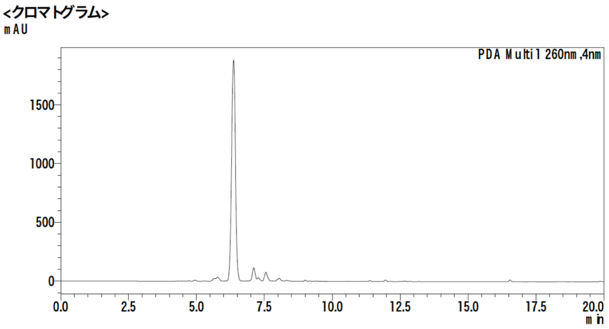

Supplementary Figure 8. HPLC analysis of PyAOP/NMM condition (entry 6).

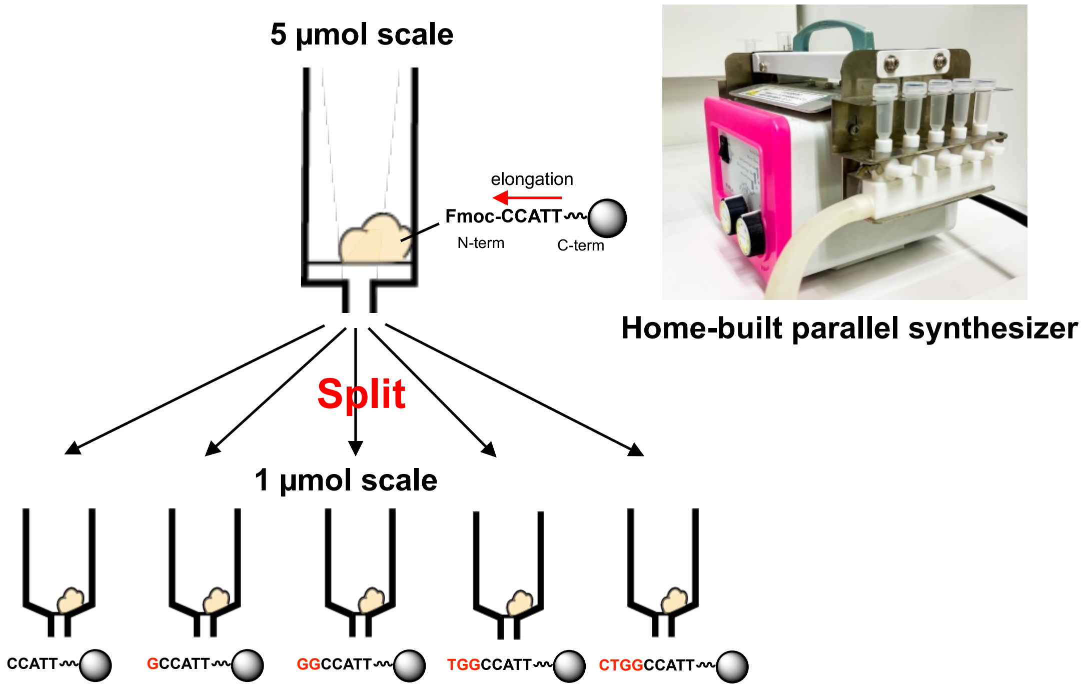

Supplementary Figure 9. PNA Synthesis Procedure using a home-built parallel PNA synthesizer.

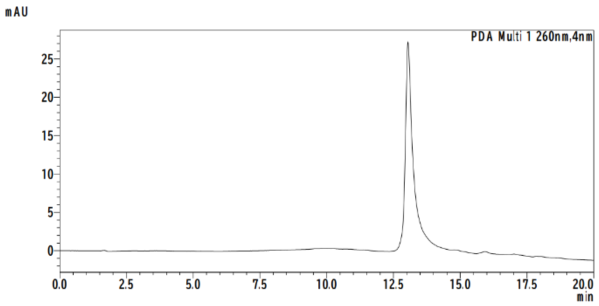

Column: COSMOSIL packed ^{5}C18-MS-II
(4.6 mm I.D. ×50 mm)
Solution A: 100 mM HFIP 8.6 mM TEA
Solution B: MeOH
T. Flow: 0.5 mL/min
5-40 % B in 20 min

MALDI-TOF-MS
Calcd: 5441.75 Found: 5464.16

mPCS1

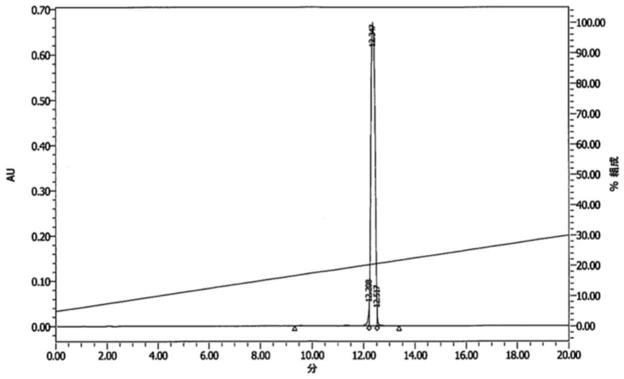

Column: X-Bridge C18
(4.6 mm I.D. ×75 mm)
Solution A: 100 mM HFIP 8 mM TEA
Solution B: MeOH
T. Flow: 1.0 mL/min
5-30 % B in 20 min

MALDI-TOF-MS
Calcd: 5130.42 Found: 5130.76

mPCS1b

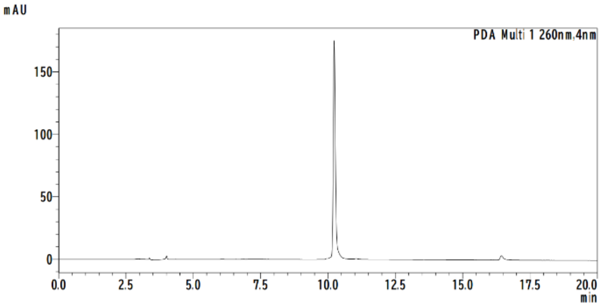

Column: COSMOSIL packed ^{5}C18-AR-II
(4.6 mm I.D. ×100 mm)
Solution A: 0.1% TFA
Solution B: 0.08% TFA in MeCN
T. Flow: 1.0 mL/min
5-40 % B in 20 min

MALDI-TOF-MS
Calcd: 2790.10   Found: 2793.90

PNA(10)-mPCS1

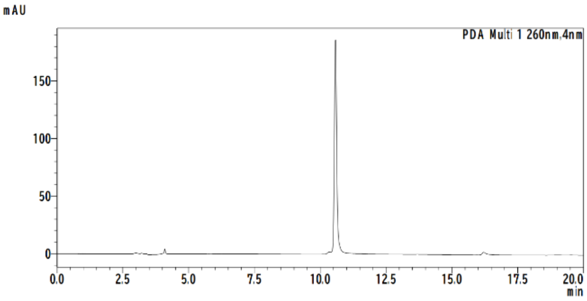

Column: COSMOSIL packed ^{5}C18-AR-II
(4.6 mm I.D. ×100 mm)
Solution A: 0.1% TFA
Solution B: 0.08% TFA in MeCN
T. Flow: 1.0 mL/min
5-40 % B in 20 min

MALDI-TOF-MS
Calcd: 3081.21   Found: 3082.62

PNA(11)-mPCS1

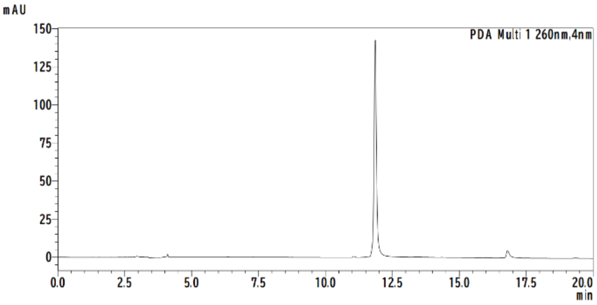

Column: COSMOSIL packed ^{5}C18-AR-II
(4.6 mm I.D. ×100 mm)
Solution A: 0.1% TFA
Solution B: 0.08% TFA in MeCN
T. Flow: 1.0 mL/min
5-40 % B in 20 min

MALDI-TOF-MS
Calcd: 3347.31   Found: 3352.23

PNA(12)-mPCS1

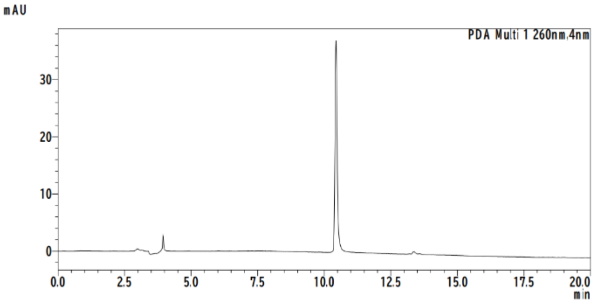

Column: COSMOSIL packed ^{5}C18-AR-II
(4.6 mm I.D. ×100 mm)
Solution A: 0.1% TFA
Solution B: 0.08% TFA in MeCN
T. Flow: 1.0 mL/min
5-40 % B in 20 min

MALDI-TOF-MS
Calcd: 2205.86   Found: 2209.45

PNA(C8)-mPCS1

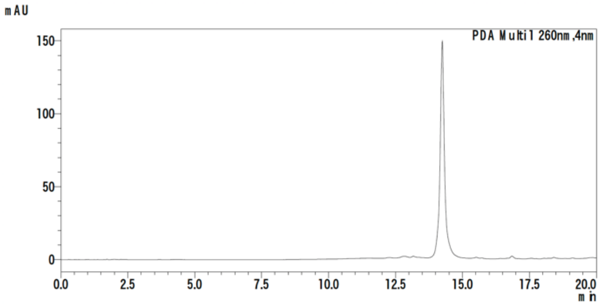

Column: COSMOSIL packed ^{5}C18-MS-II
(4.6 mm I.D. ×50 mm)
Solution A: 100 mM HFIP 8.6 mM TEA
Solution B: MeOH
T. Flow: 0.5 mL/min
5-40 % B in 20 min

MALDI-TOF-MS
Calcd: 5232.74   Found: 5230.25

hApo1

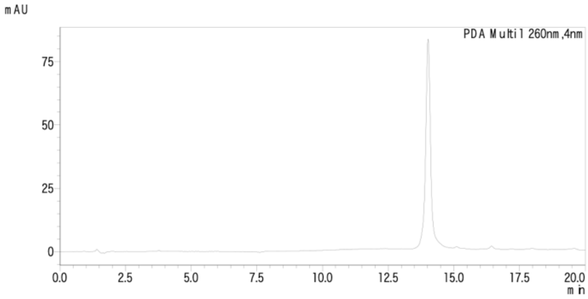

Column: COSMOSIL packed ^{5}C18-MS-II
(4.6 mm I.D. ×50 mm)
Solution A: 100 mM HFIP 8.6 mM TEA
Solution B: MeOH
T. Flow: 0.5 mL/min
5-40 % B in 20 min

MALDI-TOF-MS
Calcd:4320.44   Found: 4320.571

hApo1n

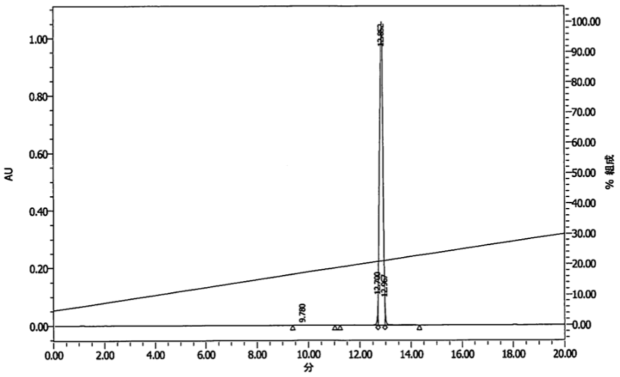

Column: X-Bridge C18
(4.6 mm I.D. ×75 mm)
Solution A: 100 mM HFIP 8 mM TEA
Solution B: MeOH
T. Flow: 1.0 mL/min
5-30 % B in 20 min

MALDI-TOF-MS
Calcd: 4893.16   Found: 4893.15

hApo1b

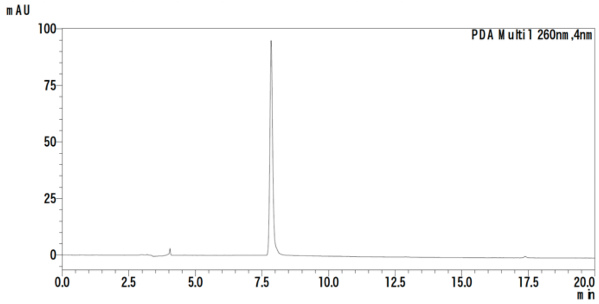

Column: COSMOSIL packed ^{5}C18-AR-II
(4.6 mm I.D. ×100 mm)
Solution A: 0.1% TFA
Solution B: 0.08% TFA in MeCN
T. Flow: 1.0 mL/min
5-40 % B in 20 min

MALDI-TOF-MS
Calcd: 1327.31   Found: 1327.33

PNA(C5)-hApo1

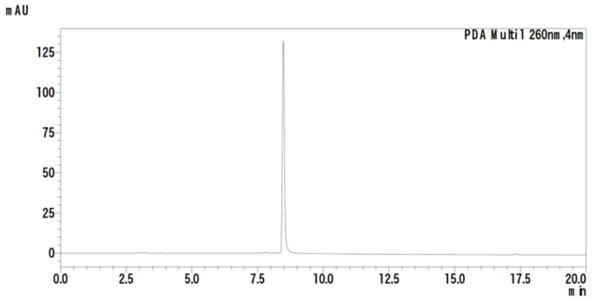

Column: COSMOSIL packed ^{5}C18-AR-II
(4.6 mm I.D. ×100 mm)
Solution A: 0.1% TFA
Solution B: 0.08% TFA in MeCN
T. Flow: 1.0 mL/min
5-40 % B in 20 min

MALDI-TOF-MS
Calcd: 1617.65 Found: 1618.47

PNA(C6)-hApo1

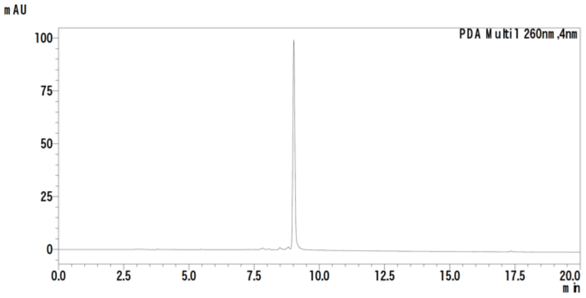

Column: COSMOSIL packed ^{5}C18-AR-II
(4.6 mm I.D. ×100 mm)
Solution A: 0.1% TFA
Solution B: 0.08% TFA in MeCN
T. Flow: 1.0 mL/min
5-40 % B in 20 min

MALDI-TOF-MS
Calcd:1909.85 Found: 1909.76

PNA(C7)-hApo1

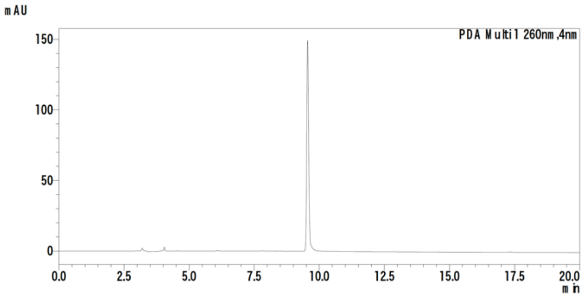

Column: COSMOSIL packed ^{5}C18-AR-II
(4.6 mm I.D. ×100 mm)
Solution A: 0.1% TFA
Solution B: 0.08% TFA in MeCN
T. Flow: 1.0 mL/min
5-40 % B in 20 min

MALDI-TOF-MS
Calcd: 2176.11 Found: 2176.97

PNA(C8)-hApo1

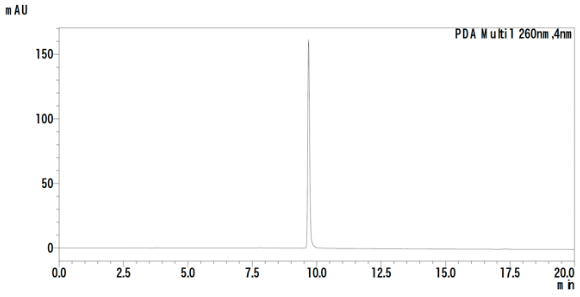

Column: COSMOSIL packed ^{5}C18-AR-II
(4.6 mm I.D. ×100 mm)
Solution A: 0.1% TFA
Solution B: 0.08% TFA in MeCN
T. Flow: 1.0 mL/min
5-40 % B in 20 min

MALDI-TOF-MS
Calcd: 2427.35 Found: 2428.25

PNA(C9)-hApo1

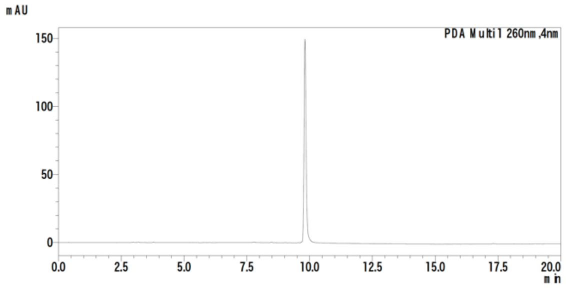

PNA(C10)-hApo1

Column: COSMOSIL packed ^{5}C18-AR-II
(4.6 mm I.D. ×100 mm)
Solution A: 0.1% TFA
Solution B: 0.08% TFA in MeCN
T. Flow: 1.0 mL/min
5-40 % B in 20 min

MALDI-TOF-MS
Calcd: 2718.63 Found: 2719.59

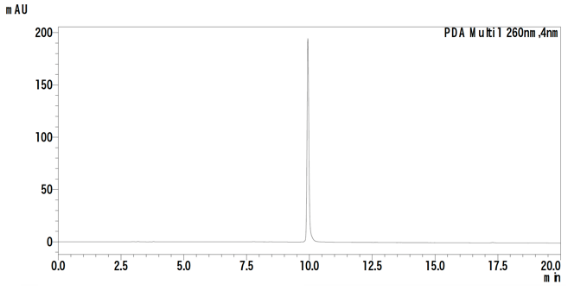

PNA(C11)-hApo1

Column: COSMOSIL packed ^{5}C18-AR-II
(4.6 mm I.D. ×100 mm)
Solution A: 0.1% TFA
Solution B: 0.08% TFA in MeCN
T. Flow: 1.0 mL/min
5-40 % B in 20 min

MALDI-TOF-MS
Calcd: 2993.90 Found: 2719.59

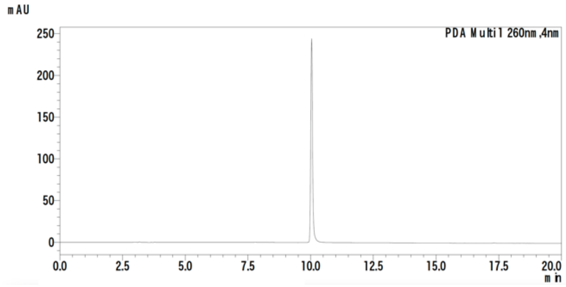

PNA(C12)-hApo1

Column: COSMOSIL packed ^{5}C18-AR-II
(4.6 mm I.D. ×100 mm)
Solution A: 0.1% TFA
Solution B: 0.08% TFA in MeCN
T. Flow: 1.0 mL/min
5-40 % B in 20 min

MALDI-TOF-MS
Calcd: 3269.17 Found: 3270.24

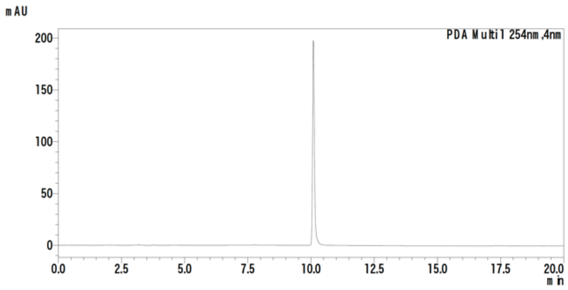

PNA(C13)-hApo1

Column: COSMOSIL packed ^{5}C18-AR-II
(4.6 mm I.D. ×100 mm)
Solution A: 0.1% TFA
Solution B: 0.08% TFA in MeCN
T. Flow: 1.0 mL/min
5-40 % B in 20 min

MALDI-TOF-MS
Calcd: 3520.42 Found: 3521.65

HPLC

Instrument : Agilent 1100 Series (Agilent Technologies)

Purity : 99.9%

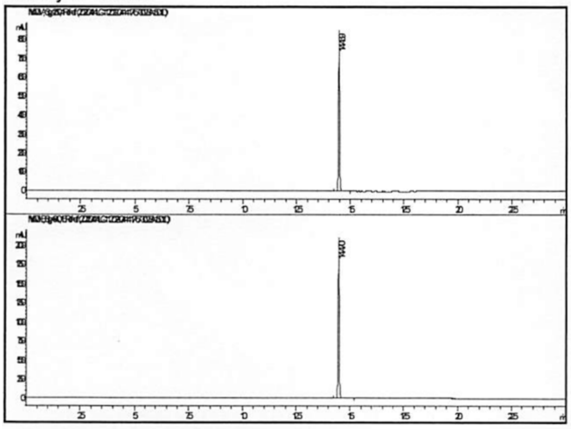

MALDI-TOF-MS
Found: 3194.6

PNA(C9)F-hApo1

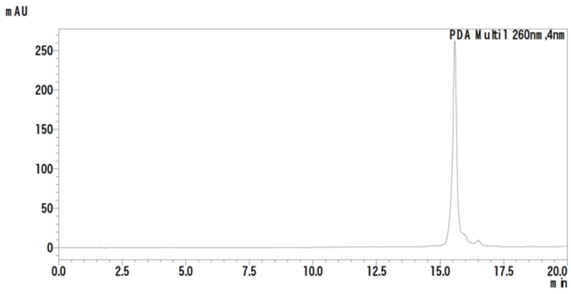

Column: COSMOSIL packed ^{5}C18-MS-II
(4.6 mm I.D. ×50 mm)
Solution A: 100 mM HFIP 8.6 mM TEA
Solution B: MeOH
T. Flow: 0.5 mL/min
5-40 % B in 20 min

MALDI-TOF-MS
Calcd: 7249.06 Found: 7246.050

mAcsl1

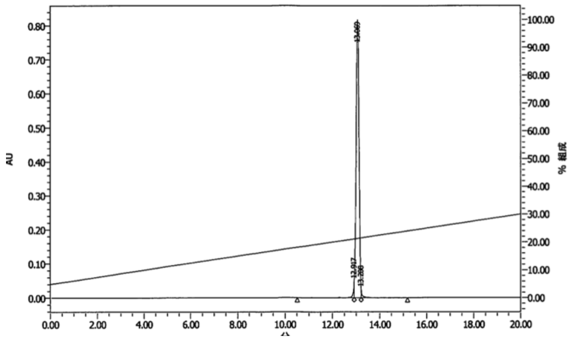

Column: X-Bridge C18
(4.6 mm I.D. ×75 mm)
Solution A: 100 mM HFIP 8 mM TEA
Solution B: MeOH
T. Flow: 1.0 mL/min
5-30 % B in 20 min

MALDI-TOF-MS
Calcd: 6454.53 Found: 6453.10

mAcsl1b

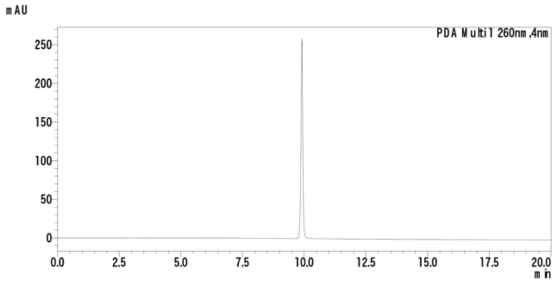

Column: COSMOSIL packed ^{5}C18-AR-II
(4.6 mm I.D. ×100 mm)
Solution A: 0.1% TFA
Solution B: 0.08% TFA in MeCN
T. Flow: 1.0 mL/min
5-40 % B in 20 min

MALDI-TOF-MS
Calcd: 3814.50 Found: 3814.52

PNA(C14)-mAcsl1

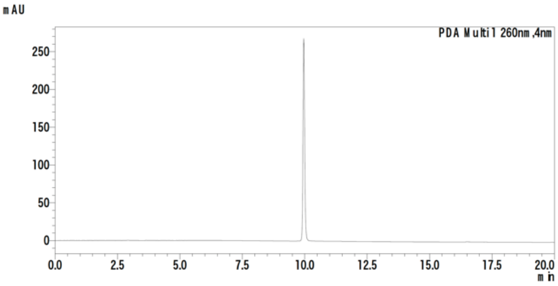

Column: COSMOSIL packed ^{5}C18-AR-II
(4.6 mm I.D. ×100 mm)
Solution A: 0.1% TFA
Solution B: 0.08% TFA in MeCN
T. Flow: 1.0 mL/min
5-40 % B in 20 min

MALDI-TOF-MS
Calcd:4105.61 Found: 4105.76

PNA(C15)-mAcsl1

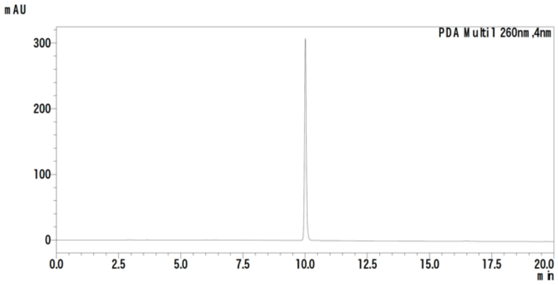

Column: COSMOSIL packed ^{5}C18-AR-II
(4.6 mm I.D. ×100 mm)
Solution A: 0.1% TFA
Solution B: 0.08% TFA in MeCN
T. Flow: 1.0 mL/min
5-40 % B in 20 min

MALDI-TOF-MS
Calcd: 4396.72 Found: 4396.87

PNA(C16)-mAcsl1

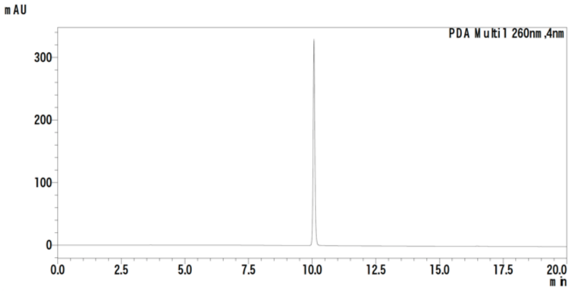

Column: COSMOSIL packed ^{5}C18-AR-II
(4.6 mm I.D. ×100 mm)
Solution A: 0.1% TFA
Solution B: 0.08% TFA in MeCN
T. Flow: 1.0 mL/min
5-40 % B in 20 min

MALDI-TOF-MS
Calcd: 4671.83 Found: 4672.15

PNA(C17)-mAcsl1

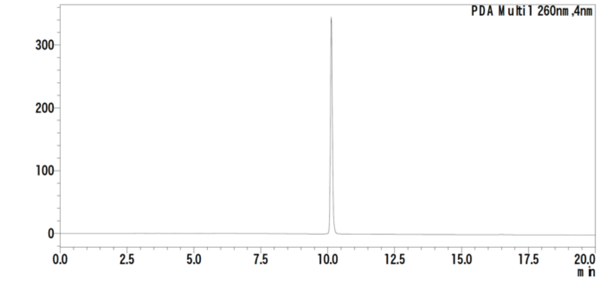

Column: COSMOSIL packed ^{5}C18-AR-II
(4.6 mm I.D. ×100 mm)
Solution A: 0.1% TFA
Solution B: 0.08% TFA in MeCN
T. Flow: 1.0 mL/min
5-40 % B in 20 min

MALDI-TOF-MS
Calcd:4962.94 Found: 4963.29

PNA(C18)-mAcsl1

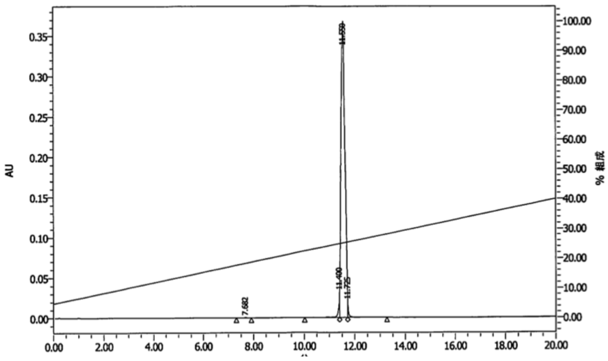

Column: X-Bridge C18
(4.6 mm I.D. ×75 mm)
Solution A: 100 mM HFIP 8 mM TEA
Solution B: MeOH
T. Flow: 1.0 mL/min
5-30 % B in 20 min

MALDI-TOF-MS
Calcd: 5002.22   Found: 4999.90

hApo1nF

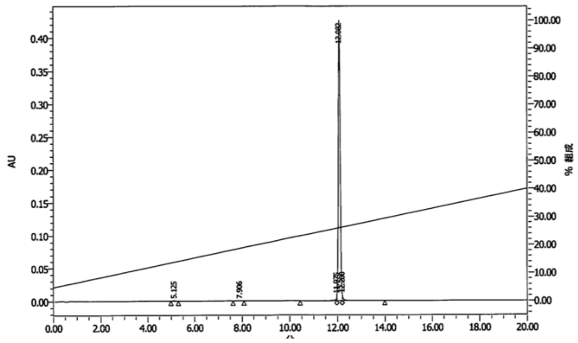

Column: X-Bridge C18
(4.6 mm I.D. ×75 mm)
Solution A: 100 mM HFIP 8 mM TEA
Solution B: MeOH
T. Flow: 1.0 mL/min
5-30 % B in 20 min

MALDI-TOF-MS
Calcd: 4656.97   Found: 4655.28

ApoB

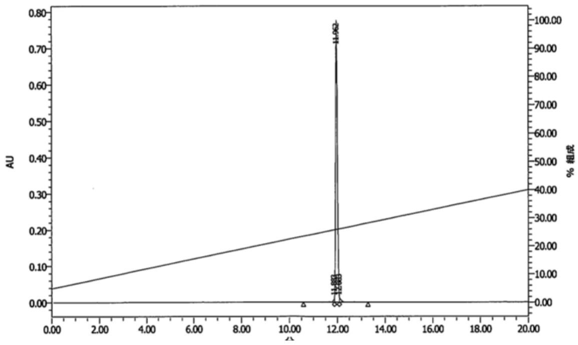

Column: X-Bridge C18
(4.6 mm I.D. ×75 mm)
Solution A: 100 mM HFIP 8 mM TEA
Solution B: MeOH
T. Flow: 1.0 mL/min
5-30 % B in 20 min

MALDI-TOF-MS
Calcd: 4696.   Found: 4693.05

Copg

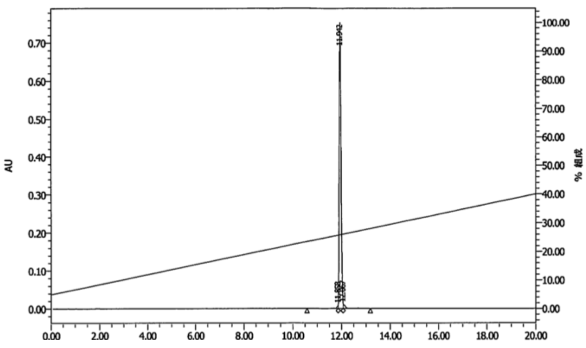

Column: X-Bridge C18
(4.6 mm I.D. ×75 mm)
Solution A: 100 mM HFIP 8 mM TEA
Solution B: MeOH
T. Flow: 1.0 mL/min
5-30 % B in 20 min

MALDI-TOF-MS
Calcd: 4696.99   Found: 4694.23

Mast

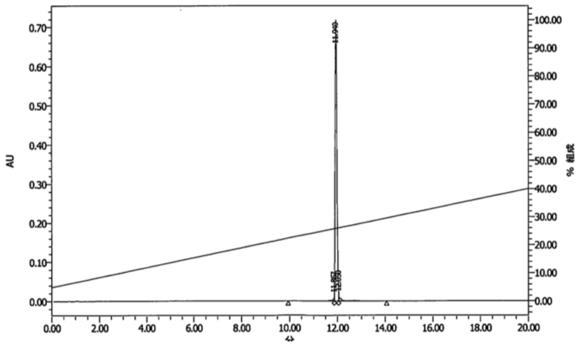

Hltf

Column: X-Bridge C18
(4.6 mm I.D. ×75 mm)
Solution A: 100 mM HFIP 8 mM TEA
Solution B: MeOH
T. Flow: 1.0 mL/min
5-30 % B in 20 min

MALDI-TOF-MS
Calcd: 4657.96 Found: 4654.85

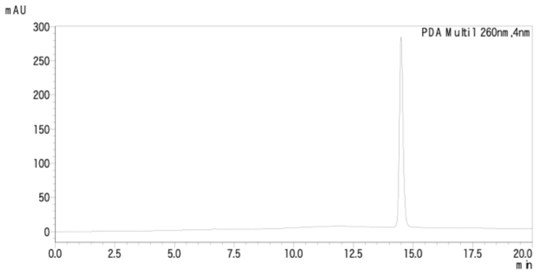

cGapmer (LNA3)

Column: COSMOSIL packed ^{5}C18-MS-II
(4.6 mm I.D. ×50 mm)
Solution A: 100 mM HFIP 8.6 mM TEA
Solution B: MeOH
T. Flow: 0.5 mL/min
5-40 % B in 20 min

MALDI-TOF-MS
Calcd: 4222.42 Found: 4220.71

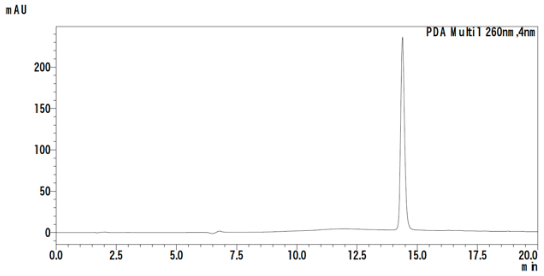

cGapmer (LNA4)

Column: COSMOSIL packed ^{5}C18-MS-II
(4.6 mm I.D. ×50 mm)
Solution A: 100 mM HFIP 8.6 mM TEA
Solution B: MeOH
T. Flow: 0.5 mL/min
5-40 % B in 20 min

MALDI-TOF-MS
Calcd: 4250.42 Found: 4250.34

Supplementary Figure 10. The purity of the materials was analyzed using analytical reverse-phase HPLC and the identification was carried out using MALDI-TOF-MS.

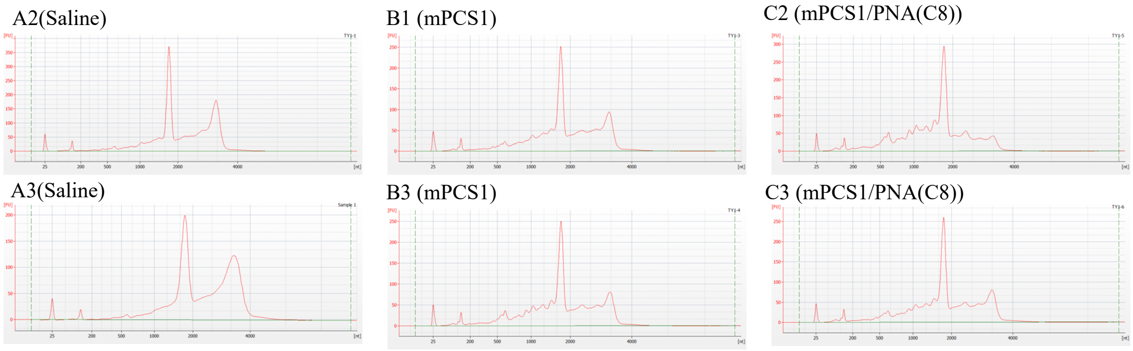

Sample	RIN
A2	7.8
A3	8.9
B1	7.1
B3	6.5
C2	5.9
C3	6.6

Supplementary Figure 11. Extracted total RNA was confirmed using bioanalyzer, and the purity was determined by RNA Integrity Number (RIN value) using a bioanalyzer.

a.

Sample number	identifier	sample_name	Title
**1**	SAMD00589509	Control-1	Saline-liver-replicate 1
**2**	SAMD00589511	Control-2	Saline-liver-replicate 2
**3**	SAMD00589513	ssASO-1	mPCS1-liver-replicate 1
**4**	SAMD00589515	ssASO-2	mPCS1-liver-replicate 2
**5**	SAMD00589517	BRO-1	BRO-liver-replicate 1
**6**	SAMD00589519	BRO-2	BRO-liver-replicate 2

b. **c.**

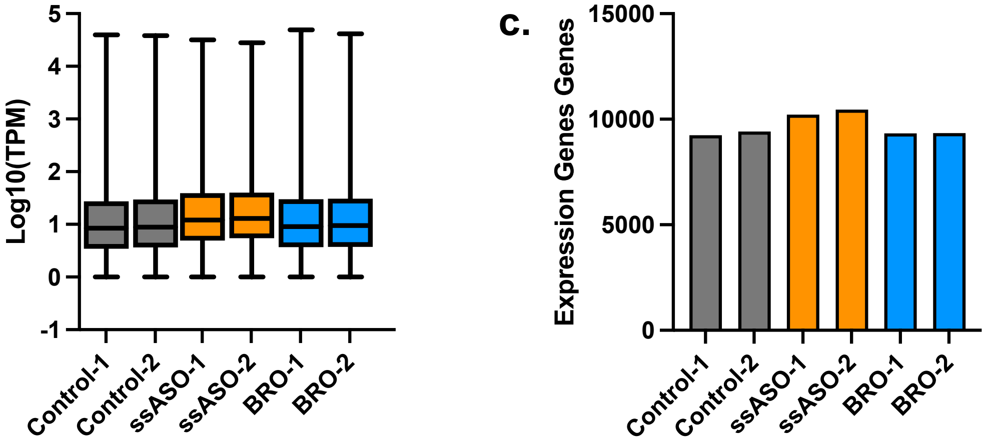

Supplementary Figure 12. **a.** Table listing names and labels of samples deposited in the DDBJ. **b.** Box-and-whisker plot illustrating the expression value (represented as

Transcripts Per Million (TPM) (>1)) for each sample. In the box-and-whisker plot, the bottom whisker denotes the minimum value, the bottom of the box indicates the first quartile, the middle line within the box represents the median value, the top of the box shows the third quartile, and the top whisker signifies the maximum value. **c.** Bar graph presenting the estimated number of expressed genes represented by the number of TPM (>1) of each sample. Each TPM value is derived from RNAseq analysis.

a.

Sample number	identifier	sample_name	Title
**1**	SAMD00589509	Control-1	Saline-liver-replicate 1
**2**	SAMD00589511	Control-2	Saline-liver-replicate 2
**3**	SAMD00589513	ssASO-1	mPCS1-liver-replicate 1
**4**	SAMD00589515	ssASO-2	mPCS1-liver-replicate 2
**5**	SAMD00589517	BRO-1	BRO-liver-replicate 1
**6**	SAMD00589519	BRO-2	BRO-liver-replicate 2

b.

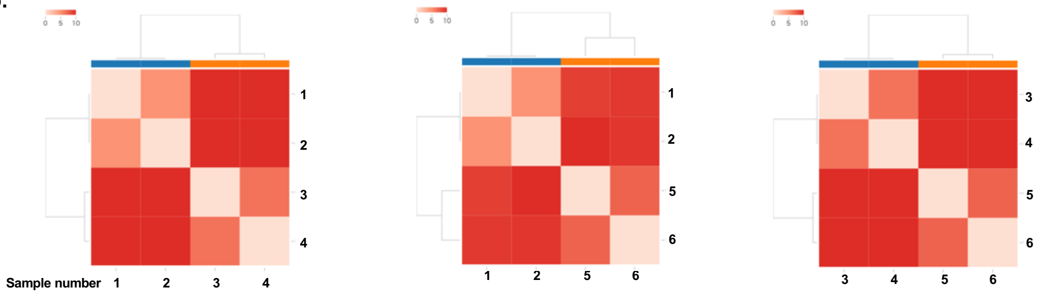

c.

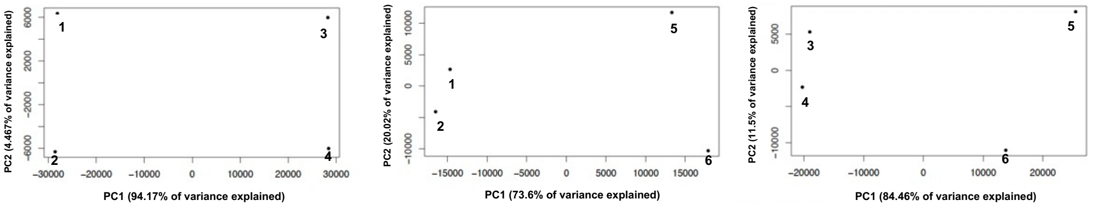

Supplementary Figure 13. **a.** List of names and labels of samples deposited in DDBJ. **b.** Heatmaps of expression similarity between samples based on the SERE coefficient.[1] **c.** PCA plots. These plots were created by re-labeling the output data from the RaNAseq analysis.

Supplementary Table 3. The number of change in gene expression as the results of introduction of **mPCS1** or **mPCS1/PNA (C8)**.

		d = 0	*d* = 1	*d* = 2	*d* > 2	Total
**# of off-target (mouse)**	**Theoretical**	5	250	6643	-	-
	**Expressed**	5	~219	~5526	~12315	~18065
**# of DEG (Saline vs ssASO)**	**Up**	0	6	255	695	956
	**Down**	4	50	467	666	1187
	**Total**	4	56	722	1361	2143
**# of DEG (Saline vs BRO)**	**Up**	0	0	81	220	301
	**Down**	2	15	107	137	261
	**Total**	2	15	188	357	562

d indicates the total number of mismatches, insertions, or deletions between the ASO and complementary RNA.

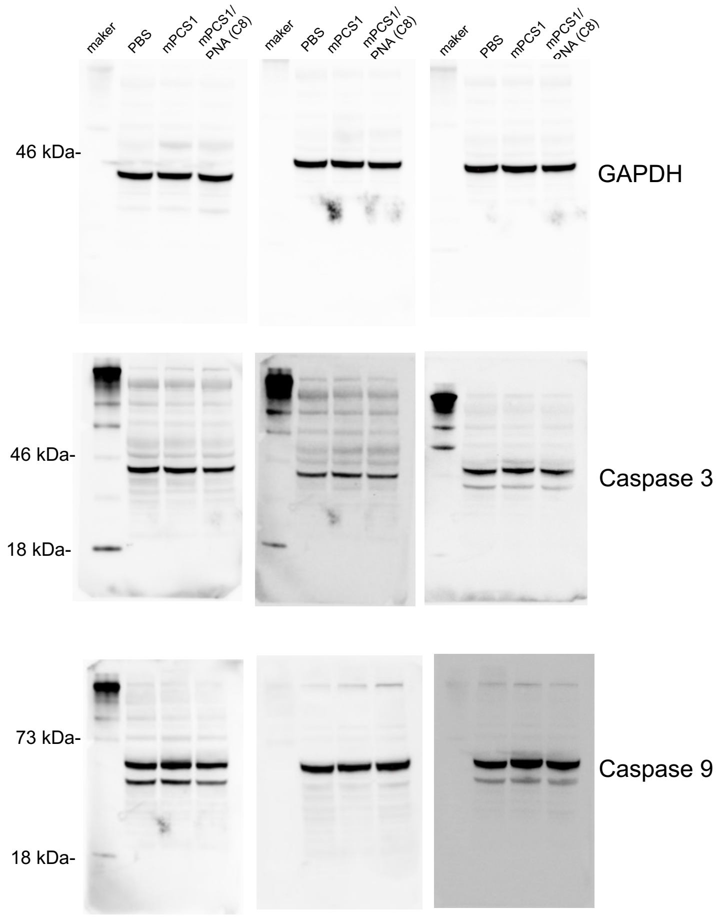

Supplementary Figure 14. Full length blot images presented in **Figure 3g** (*n* = 3 biologically independent samples).

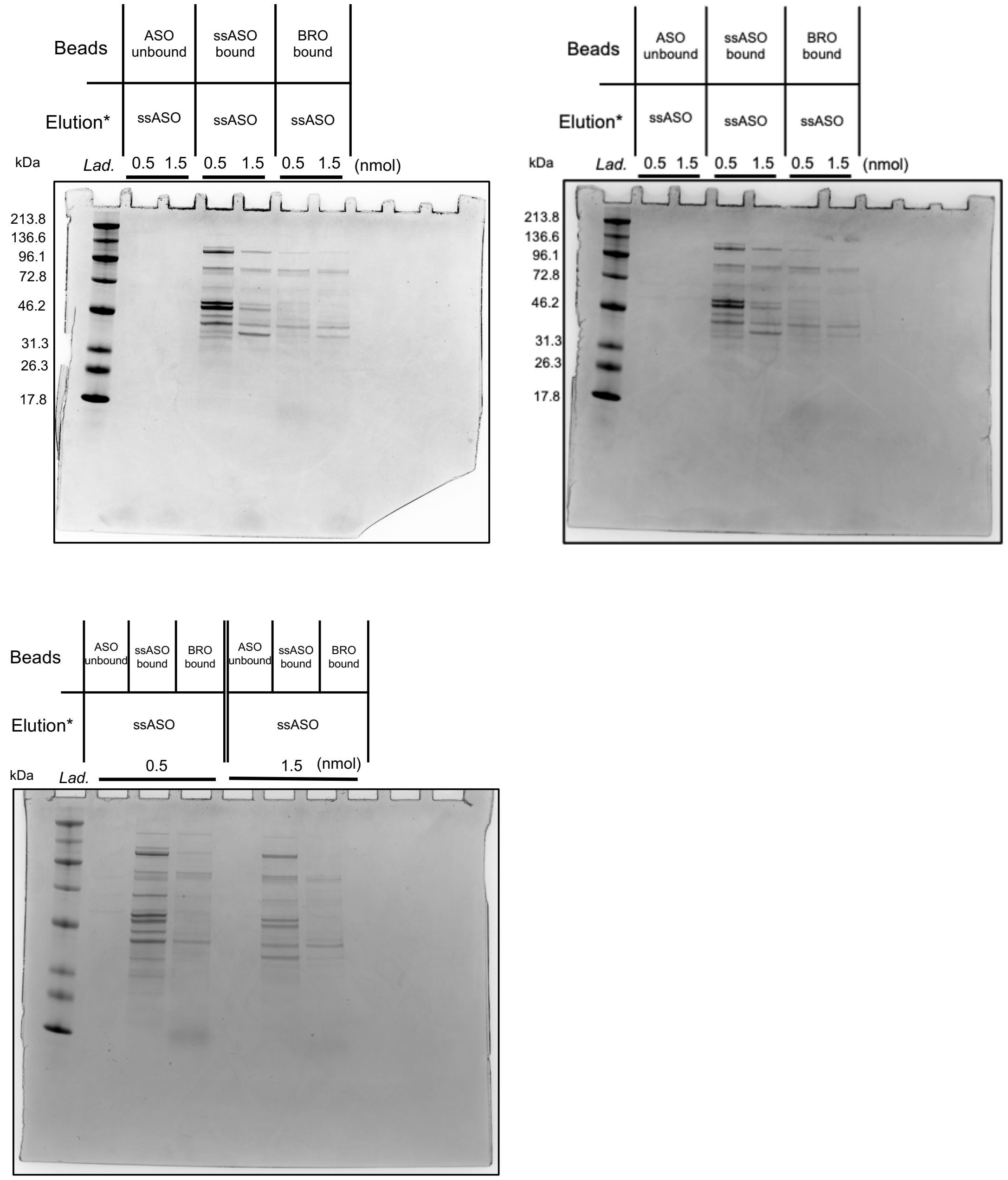

Supplementary Figure 15. CBB staining of binding proteins that isolated from liver lysate with capture **mPCS1b** or **mPCS1b/PNA(C8)** BRO and eluted by **mPCS1n** (*n* = 3 biologically independent samples).

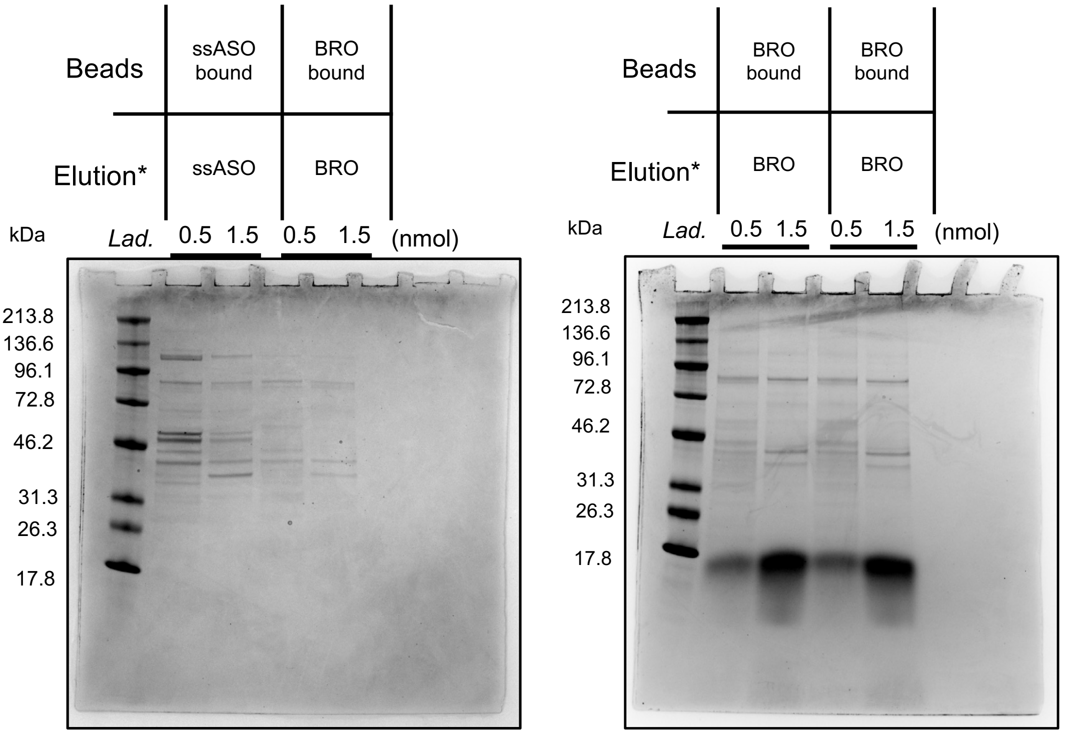

N=3, independent experiments

Supplementary Figure 16. CBB staining of binding proteins that isolated from liver lysate with capture **mPCS1b** or **mPCS1b/PNA(C8)** BRO and eluted by **mPCS1n** or **mPCS1b/PNA(C8)** BRO (*n* = 3 biologically independent samples).

.

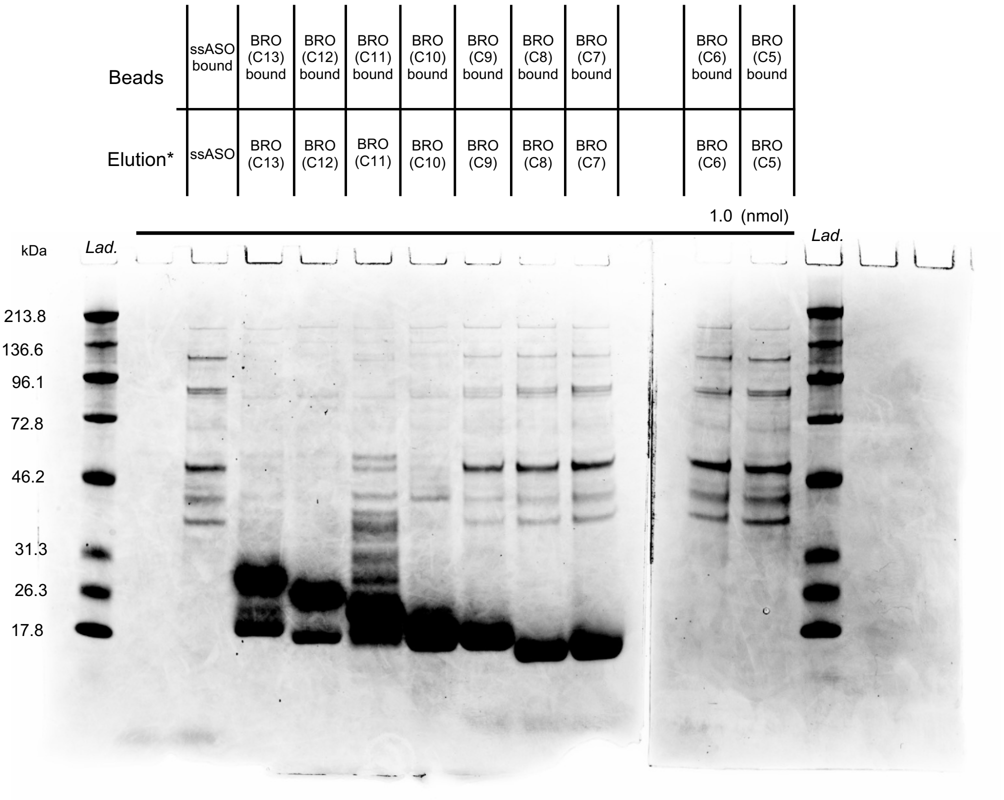

Supplementary Figure 17. CBB staining of binding proteins that isolated from liver lysate with capture **hApo1b** or **hApo1b/PNA(C5-C13)** BRO and eluted by **hApo1n or hApo1b/PNA(C5-C13)** BRO (*n* = 2 biologically independent samples).

Supplementary Table 4. Sequences and chemistries in this study.

ID		Sequence (5'-3')		target
mPCS1	5'	YYA^C^A^c^c^a^a^g^t^t^c^T^C^c	3'	Pcsk9 (mouse)
mPCS1n		A^C^A^c^c^a^a^g^t^t^c^T^C^c		
mPCS1b		b^A^C^A^c^c^a^a^g^t^t^c^T^C^c		
mPCS1f		A^C^A^c^c^a^a^g^t^t^c^T^C^c^N(6)-Alexa647		
PNA(12)-mPCS1	C-term	*TGGTTCAAGAGG*	N-term	
PNA(11)-mPCS1		*GGTTCAAGAGG*		
PNA(10)-mPCS1		*GTTCAAGAGG*		
PNA(C8)-mPCS1		*TGTGGTTC*		
cRNA(14)-mPCS1	3'	rUrGrUrGrGrUrUrCrArArGrArGrG	5'	
hApo1	5'	YYA^A^t^g^g^c^c^a^^g^c^T^T^G	3'	ApoB (human/mouse)
hApo1n		A^A^t^g^g^c^c^a^^g^c^T^T^G		
hApo1b		b^A^A^t^g^g^c^c^a^^g^c^T^T^G		
PNA(C5)-hApo1	C-term	*TTACC*	N-term	
PNA(C6)-hApo1		*TTACCG*		
PNA(C7)-hApo1		*TTACCGG*		
PNA(C8)-hApo1		*TTACCGGT*		
PNA(C9)-hApo1		*TTACCGGTC*		
PNA(C10)-hApo1		*TTACCGGTCG*		
PNA(C11)-hApo1		*TTACCGGTCGA*		
PNA(C12)-hApo1		*TTACCGGTCGAA*		
PNA(C13)-hApo1		*TTACCGGTCGAAC*		
PNA(C9)F-hApo1		(Cy5)Lys-*TTACCGGTC*		
cRNA(13)-hApo1	3'	rUrUrArCrCrGrGrUrCrGrArArC	5'	
hApo1nF	5'	TAMRA-N(H)^A^A^t^g^g^c^c^a^g^c^T^T^G	3'	
cGapmer (LNA3)	3'	T^t^a^c^c^g^g^t^c^g^a^A^C	5'	
cGapmer (LNA4)		T^T^a^c^c^g^g^t^c^g^a^A^C		
ApoB		QrUrUrArCrCrGrGrUrCrGrArArC		
Copg		QrUrUrArCrCrGrGrGrCrGrArArC		
Mast2		QrUrUrArCrCrGrGrUrGrGrArArC		
Hltf		QrUrUrArCrCrGrGrUrUrGrArArC		
Acsl1	5'	YYYC^T^C^c^a^t^g^a^c^a^c^a^g^c^a^T^T^a	3'	Acsl1 (human/mouse)
Acsl1n		C^T^C^c^a^t^g^a^c^a^c^a^g^c^a^T^T^a		
Acsl1b		b^C^T^C^c^a^t^g^a^c^a^c^a^g^c^a^T^T^a		
pPNA(C14)-Acsl1	C-term	TACTGTGTCGTAAT	N-term	
pPNA(C15)-Acsl1		GTACTGTGTCGTAAT		
pPNA(C16)-Acsl1		GGTACTGTGTCGTAAT		
pPNA(C17)-Acsl1		AGGTACTGTGTCGTAAT		
pPNA(C18)-Acsl1		GAGGTACTGTGTCGTAAT		
cRNA(18)-Acsl1	3'	rGrArGrGrUrArCrUrGrUrGrUrCrGrUrArArU	5'	

N indicates LNA; n indicates DNA; *N* indicates PNA; rN indicates RNA; ^ indicates PS linkage; Y indicates GalNAc$_{APD}$; b indicates biotin; N(6) indicates amino C6 linker; N(H) indicates ssH amino linker.

Supplementary Table 5. Antibodies information in this study.

Antibodies	Source	Catalog Number	Lot Number	Dilution	Clone No.
Anti-nmr55/p54nrb	abcam	ab70335	GR3404136-1	1:200	
Goat Anti-Rabbit IgG H+L (Alexa Fluor 488)	abcam	ab150077	GR3376391-4	1:1000	
Anti-GAPDH	proteintech	60004-1-lg	10025237	1:10000	1E6D9
Anti-Caspase 3/p17/p19	proteintech	66470-2-lg	10021291	1:3000	2G4B2
Anti-Caspase 9/p35/p10	proteintech	66169-1-lg	10003480	1:1000	1B7G2
Anti-IgG, Mouse, Goat-Poly, HRP	RSD	HAF007	FIM3120081	1:1000	

Supplementary Table 6. Primer-probe sets for qRT-PCR.

Primer-probe sets	Sequence (5'-3')
human ApoB	Fw: TTCTCAAGAGTTACAGCAGATCCA<br>Rv: TGGAAGTCCTTAAGAGCAACTAACA
human GAPDH	Fw: GCACCGTCAAGGCTGAGAAC<br>Rv: TGGTGAAGACGCCAGTGGA
human HLTF	Fw: CCTGTGCCGTTTTCTTGCTC<br>Rv: CCCTGCAGAAAGGTCTTGGT
human MAST	Fw: TGACTTTCGCTGAGAACCCC<br>Rv: TCTTCAGCAGAGTGGCACAG
human COPG	Fw: CCATGCATTGGGAGTCCTGT<br>Rv: ACTGGCAATTCGGATCAGCA
human MLKL	Fw: TCACACTTGGCAAGCGCATGGT<br>Rv: GTAGCCTTGAGTTACCAGGAAGT
mouse ApoB	Fw: TCCTCGGTGAGTTCAATGACTTTC<br>Rv: TGGACCTGCTGTAGCTTGTAGGA
mouse Pcsk9	Fw: TCAGTTCTGCACACCTCCAG<br>Rv: GGGTAAGGTGCGGTAAGTCC
mouse Acsl1	Fw: AGGTGCTTCAGCCCACCATC<br>Rv: AAAGTCCAACAGCCATCGCTTC
mouse Cdkn1a	Fw: TCGCTGTCTTGCACTCTGGTGT<br>Rv: CCAATCTGCGCTTGGAGTGATAG
mouse MLKL	Fw: CTGAGGGAACTGCTGGATAGAG<br>Rv: CGAGGAAACTGGAGCTGCTGAT
mouse IL18	Fw: TTCTGCAACCTCCAGCATCA<br>Rv: AGTGAAGTCGGCCAAAGTTGTCT
mouse TNFα	Fw: TATGGCCCAGACCCTCACA<br>Rv: GGAGTAGACAAGGTACAACCCATG
mouse IL4	Fw: ACGGAGATGGATGTGCCAAAC<br>Rv: AGCACCTTGGAAGCCCTACAGA
mouse IL6	Fw: CAACGATGATGCACTTGCAGA<br>Rv: CTCCAGGTAGCTATGGTACTCCAGA
mouse IFNα	Fw: CTGTGCTTTCCTGATGGTCCTG<br>Rv: GGAATCCAAAGTCCTTCCTGTCCT
mouse IFNγ	Fw: CGGCACAGTCATTGAAAGCCTA<br>Rv: GTTGCTGATGGCCTGATTGTC
mouse GAPDH	Fw: TGTGTCCGTCGTGGATCTGA<br>Rv: TTGCTGTTGAAGTCGCAGGAG

Supplementary Table 7. HE staining procedure

No.	material	Time
1	Xylene	10min
2	Xylene	5min
3	Xylene	5min
4	100%E-Alc	3min
5	100%E-Alc	3min
6	100%E-Alc	3min
7	70%E-Alc	3min
8	Tap-Water	4min
9	D.W	10sec
10	Hematoxylin 3G	5min
11	Tap-Water	3min
12	Hcl/E-Alc	10sec
13	Tap-Water	10min
14	D.W	10sec
15	70%E-Alc	2min
16	80%E-Alc	2min
17	Eosin	5min
18	Eosin	5min
19	95%E-Alc	1min
20	100%E-Alc	5min
21	100%E-Alc	5min
22	100%E-Alc	5min
23	Xylene	5min
24	Xylene	5min
25	Xylene	5min

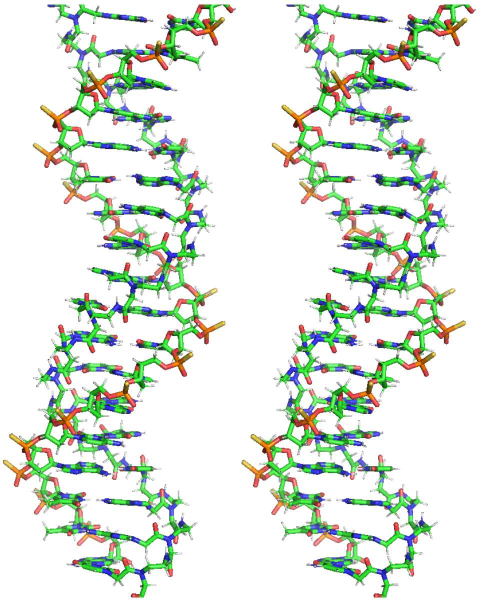

Supplementary Figure 18. Stereoscopic view of ASO and parallel PNA duplex model

Supplementary Table 8. Database Search Results for Potential Target Pre-mRNAs of **Acsl1** ASO

mismatches or gaps	potential targets for **Acsl1** ASO	Number of hits
0	Acsl1	1
1	–	0
2	Vmn2r82, Vmn2r59, Nme9, Lipi, Zap70, Dchs2, Sptbn5, Pate6, Rimklb, Vmn2r81, 1600029I14Rik	11

Database: Mouse spliced RNA, RefSeq curated on GRCm39/mm39, D3G 23.02 (Feb, 2023)
Site: https://gggenome.dbcls.jp

Supplementary References

1. Schulze, S. K., Kanwar, R., Gölzenleuchter, M., Therneau, T. M. & Beutler, A. S. SERE: Single-parameter quality control and sample comparison for RNA-Seq. *BMC Genomics* **13**, 524 (2012).
