# Supplementary material for: Dynamic and static control of the off-target interactions of antisense oligonucleotides using toehold chemistry
Source: Nat Commun. 2023 Dec 2;14:7972. doi: 10.1038/s41467-023-43714-0 (PMC10693639; doi:10.1038/s41467-023-43714-0)
Supplement: Supplementary file 3 — Description of Additional Supplementary Files [file 41467_2023_43714_MOESM3_ESM.pdf]

<u>Description of Additional Supplementary Files</u>

File Name: Supplementary Data 1

Description: Detailed of binding proteins presented in Figure 5d.

File Name: Supplementary Data 2

Description: GSEA analysis (BRO(mPCS1/PNA(C8)) VS mPCS1).

File Name: Supplementary Data 3

Description: The coordinates of ASO and parallel PNA duplex model
