# Supplementary material for: Dynamic and static control of the off-target interactions of antisense oligonucleotides using toehold chemistry
Source: Nat Commun. 2023 Dec 2;14:7972. doi: 10.1038/s41467-023-43714-0 (PMC10693639; doi:10.1038/s41467-023-43714-0)
Supplement: Supplementary file 7 — Reporting Summary [file 41467_2023_43714_MOESM7_ESM.pdf]

nature portfolio

Corresponding author(s): Tsuyoshi Yamamoto

Last updated by author(s): Nov 8, 2023

Reporting Summary

Nature Portfolio wishes to improve the reproducibility of the work that we publish. This form provides structure for consistency and transparency in reporting. For further information on Nature Portfolio policies, see our Editorial Policies and the Editorial Policy Checklist.

Statistics

For all statistical analyses, confirm that the following items are present in the figure legend, table legend, main text, or Methods section.

n/a	Confirmed	
☐	☒	The exact sample size (*n*) for each experimental group/condition, given as a discrete number and unit of measurement
☐	☒	A statement on whether measurements were taken from distinct samples or whether the same sample was measured repeatedly
☐	☒	The statistical test(s) used AND whether they are one- or two-sided<br>*Only common tests should be described solely by name; describe more complex techniques in the Methods section.*
☒	☐	A description of all covariates tested
☐	☒	A description of any assumptions or corrections, such as tests of normality and adjustment for multiple comparisons
☐	☒	A full description of the statistical parameters including central tendency (e.g. means) or other basic estimates (e.g. regression coefficient) AND variation (e.g. standard deviation) or associated estimates of uncertainty (e.g. confidence intervals)
☐	☒	For null hypothesis testing, the test statistic (e.g. *F*, *t*, *r*) with confidence intervals, effect sizes, degrees of freedom and *P* value noted<br>*Give P values as exact values whenever suitable.*
☒	☐	For Bayesian analysis, information on the choice of priors and Markov chain Monte Carlo settings
☒	☐	For hierarchical and complex designs, identification of the appropriate level for tests and full reporting of outcomes
☒	☐	Estimates of effect sizes (e.g. Cohen's *d*, Pearson's *r*), indicating how they were calculated

Our web collection on statistics for biologists contains articles on many of the points above.

Software and code

Policy information about availability of computer code

Data collection	Detailed information of data collection was included in the manuscript under section" Materials and Methods"<br>Serum AST/ALT was measured using a Fuji dry-chem slide GPT/ALT-PIII or outsourced to Oriental Yeast Co., Ltd. (Tokyo, Japan). Measurements of absorbance (for WST-8 assay and total cholesterol levels) and luminescence (for caspases 3/7, 8 and 9 assay) were collected by a multimode microplate reader (Cytation3, Agilent Technologies).<br>qRT-PCR data was collected by Applied Biosystems StepOnePlus Real-Time PCR system (Applied Biosystems).<br>Confocal images were collected by confocal laser scanning microscopy (LSM710 microscope, Carl Zeiss Co., Ltd.).<br>Gel and blot images were visualized by Bio-Rad Molecular Imager ChemiDoc Touch.<br>Measurement of fluorescence (for PI staining and JC-1 MitoMP detection assay) was visualized using a fluorescence microscope (Axio Vert.Al, Carl Zeiss Co., Ltd.).<br>Fluorescence intensity for in-cuvette FRET assay was measured using a fluorometer (FP-8200, JASCO)<br>UV melting experiments were performed using a Shimadzu UV-1850 spectrophotometer equipped with a TMSPC-8 Tm analysis system (Shimadzu)<br>RNA-seq samples were prepared using total RNA with high RIN from mouse livers with Collibri(TM) 3'mRNA Library Prep Kit for Illumina(TM) Systems (Life Technologies, # A38110024), and sequencing data were obtained using Novaseq 6000.<br>The Biodistribution for in-vivo imaging study was visualized using an IVIS Lumina II imaging system (Caliper Life Science; excitation filter, 640 nm; emission filter, Cy5.5., exposure time= 5 s).<br>The peptide fragment of in-gel digestion was analyzed using MS/MS instrument (Q-Exactive series, Thermo Fisher Scientific) equipped with a nano-LC system (EASY-nLCTM, Thermo Fisher Scientific)
Data analysis	qRT-PCR data was analyzed by Bio-Rad CFX Maestro 1.1 (version 4.1.2433.1219).

Data analysis	Fluorescence images were analyzed by ZEN software 3.3. 89. 0000 (blue edition).<br>Confocal images were analyzed by ZEN software 3.5. 093. 00001 (blue edition).<br>Created Fastq files were processed on the RaNA-seq website (https://ranaseq.eu), and DE and functional enrichment analyses were performed with DESeq2 using the Wald test (cutoff = 0.05).<br>wPGSA was performed using RNA-seq fold change (FC) data at http://wpgsa.org.<br>Statistical analyses were performed using GraphPad Prism 9 software (Version 9.5.1).<br>Gel and blot images were analyzed using Bio-Rad Molecular Imager ChemiDoc Touch software (version 2.4.0.03).

For manuscripts utilizing custom algorithms or software that are central to the research but not yet described in published literature, software must be made available to editors and reviewers. We strongly encourage code deposition in a community repository (e.g. GitHub). See the Nature Portfolio guidelines for submitting code & software for further information.

Data

Policy information about availability of data

All manuscripts must include a data availability statement. This statement should provide the following information, where applicable:

- Accession codes, unique identifiers, or web links for publicly available datasets
- A description of any restrictions on data availability
- For clinical datasets or third party data, please ensure that the statement adheres to our policy

All information regarding access to the primary and reference datasets from this study is provided in the main text, Supplementary Figures and Tables. The RNAseq data generated in this study have been deposited in the DDBJ Sequence Read Archive under DRA Accession (DRA016038) [https://ddbj.nig.ac.jp/resource/sra-submission/DRA016038], and are publicly available. Source data are provided with this paper.

Research involving human participants, their data, or biological material

Policy information about studies with human participants or human data. See also policy information about sex, gender (identity/presentation), and sexual orientation and race, ethnicity and racism.

Reporting on sex and gender	N/A
Reporting on race, ethnicity, or other socially relevant groupings	N/A
Population characteristics	N/A
Recruitment	N/A
Ethics oversight	N/A

Note that full information on the approval of the study protocol must also be provided in the manuscript.

Field-specific reporting

Please select the one below that is the best fit for your research. If you are not sure, read the appropriate sections before making your selection.

☒ Life sciences ☐ Behavioural & social sciences ☐ Ecological, evolutionary & environmental sciences

For a reference copy of the document with all sections, see nature.com/documents/nr-reporting-summary-flat.pdf

Life sciences study design

All studies must disclose on these points even when the disclosure is negative.

Sample size	No sample-size calculation was performed. Sample sizes for all animals studies in the manuscript were listed in figure legend, maintext, or methods section. A N of 3 or more animals were used for all studies. Reference paper for the determination of sample size is follows: Nat Biotechnol 37, 640–650 (2019). https://doi.org/10.1038/s41587-019-0106-2.
Data exclusions	No dataset was excluded, except for one replicate identified as an outlier by both the Grubbs' test (Alpha = 0.05) and the ROUT method (Q = 1%) using GraphPad software in Figure 4d.
Replication	All experimental findings were replicated as described in the figure captions; note that Fig. 5c, 5e, and Fig. 2l, 4e, 7f are based on limited trials with n=2 and n=1 biologically independent samples, respectively. In general, the reproducibility and generality of the claims made in this study are also supported by the consistency of results from systematic comparisons of BROS with different but similar BSs and from analogous studies in ASOs with different sequences and compositions.
Randomization	For all experiments, samples/animals were assigned randomly to experimental and control groups
Blinding	Experiments were conducted without blinding, except for the following: serum, RNA, and pathological samples were blinded and forwarded

Blinding	to the respective analysts, who performed measurements of AST/ALT, RNAseq data acquisition, and pathological analysis.

Reporting for specific materials, systems and methods

We require information from authors about some types of materials, experimental systems and methods used in many studies. Here, indicate whether each material, system or method listed is relevant to your study. If you are not sure if a list item applies to your research, read the appropriate section before selecting a response.

Materials & experimental systems

n/a	Involved in the study	
☐	☒	Antibodies
☐	☒	Eukaryotic cell lines
☒	☐	Palaeontology and archaeology
☐	☒	Animals and other organisms
☒	☐	Clinical data
☒	☐	Dual use research of concern
☒	☐	Plants

Methods

n/a	Involved in the study	
☒	☐	ChIP-seq
☒	☐	Flow cytometry
☒	☐	MRI-based neuroimaging

Antibodies

Antibodies used

Anti-nmr55/p54nrb: abcam ab70335; Lot GR3404136-1; Dilution IF 1:200
Goat Anti-Rabbit IgG H+L (Alexa Fluor 488): abcam ab150077; Lot GR3376391-4; Dilution IF 1:1000
Anti-GAPDH: proteintech 60004-1-lg; Lot 10025237; Dilution WB 1:10000
Anti-Caspase 3/p17/p19: proteintech 66470-2-lg; Lot 10021291; Dilution WB 1:3000
Anti-Caspase 9/p35/p10: proteintech 66169-1-lg; Lot 10003480; Dilution WB 1:1000
Anti-IgG, Mouse, Goat-Poly, HRP: RSD HAF007; Lot FIM3120081; Dilution WB 1:1000

Validation

Antibodies were validated in this manuscript or in the previous publications including:
-Anti-nmr55/p54nrb: Rabbit polyclonal to nmt55 / p54nrb is used by 21 references on supplier website; https://www.abcam.co.jp/products/primary-antibodies/nmt55--p54nrb-antibody-ab70335.html
(Ex.) Ding, H et al. NONO promotes hepatocellular carcinoma progression by enhancing fatty acids biosynthesis through interacting with ACLY mRNA. Cancer Cell Int, 20: 425 (2020).

-Anti-Caspase 3/p17/p19: mouse monoclonal (Clone No.: 2G4B2) to caspase 3/p17/p19 is used by 183 references on supplier website; https://www.ptglab.co.jp/products/CASP3-Antibody-66470-2-Ig.htm
(Ex.) Zhao B, Xu P, Rowlett CM, et al. The molecular basis of tight nuclear tethering and inactivation of cGAS. Nature. 2020;587(7835):673-677.
(Ex.) Bi QC, Tang JJ, Zhao J, et al. Sevelamer arsenite nanoparticle as a Pi-responsive drug carrier and embolic agent for chemoembolization. Drug Deliv. 2022;29(1):1447-1456.

-Anti-Caspase 9/p35/p10: mouse monoclonal (Clone No.: 1B7G2) to caspase 9/p35/p10 is used by 86 references on supplier website; https://www.ptglab.co.jp/products/Caspase-9-Antibody-66169-1-Ig.htm
(Ex.) Huang Q, Zhan L, Cao H, et al. Increased mitochondrial fission promotes autophagy and hepatocellular carcinoma cell survival through the ROS-modulated coordinated regulation of the NFKB and TP53 pathways. Autophagy. 2016;12(6):999-1014.
(Ex.) Mo D, Tian W, Zhang HN, et al. Cardioprotective effects of galectin-3 inhibition against ischemia/reperfusion injury. Eur J Pharmacol. 2019;863:172701.

-Anti-GAPDH: mouse monoclonal (Clone No.: 1E6D9) to GAPDH is used by 8445 references on suppliner website; https://www.ptglab.co.jp/products/GAPDH-Antibody-60004-1-Ig.htm
(Ex.) Zhou R, Wang G, Li Q, et al. A signalling pathway for transcriptional regulation of sleep amount in mice. Nature. 2022;612(7940):519-527.

In addition, validation statement can be found on manufacturer's website with provided catalog number.

Eukaryotic cell lines

Policy information about cell lines and Sex and Gender in Research

Cell line source(s)	Huh-7 cells, a human hepatoma cell line, were purchased from JCRB Cell Bank.
Authentication	None of the cell lines used were authenticated.
Mycoplasma contamination	Cell lines were not tested for Mycoplasma contamination.
Commonly misidentified lines (See ICLAC register)	No commonly misidentified cell lines were used.

Animals and other research organisms

Policy information about studies involving animals; ARRIVE guidelines recommended for reporting animal research, and Sex and Gender in Research

Laboratory animals	Male C57BL/6J mice and Balb/cSlc-nu/nu mice aged 6-8 weeks were purchased from SLC Japan (Tokyo, Japan). Mice were acclimated for 1~2 weeks and then all experiments commenced when the animals were 6–8 weeks old.
Wild animals	No wild animals were used in this study.
Reporting on sex	Sex was not considered in the study design.
Field-collected samples	No field collected samples were used in this study.
Ethics oversight	All animal experiments were performed in accordance with the guidelines for animal experimentation of Nagasaki University (Nagasaki, Japan), with the consent of the Animal Care Ethics Committee (approval number:1911011572-6).

Note that full information on the approval of the study protocol must also be provided in the manuscript.

Plants

Seed stocks	N/A
Novel plant genotypes	N/A
Authentication	N/A
